# Plasma protein-based signature predicts distant metastasis and induction chemotherapy benefit in Nasopharyngeal Carcinoma

**DOI:** 10.7150/thno.47882

**Published:** 2020-08-01

**Authors:** Yelin Liang, Junyan Li, Qian Li, Linglong Tang, Lei Chen, Yanping Mao, Qingmei He, Xiaojing Yang, Yuan Lei, Xiaohong Hong, Yin Zhao, Shiwei He, Ying Guo, Yaqin Wang, Panpan Zhang, Na Liu, Yingqin Li, Jun Ma

**Affiliations:** 1 Department of Radiation Oncology, State Key Laboratory of Oncology in South China, Collaborative Innovation Center of Cancer Medicine, Guangdong Key Laboratory of Nasopharyngeal Carcinoma Diagnosis and Therapy, Sun Yat-sen University Cancer Center, Guangzhou, 510060, P.R. China.; 2 Clinical Trials Centre, State Key Laboratory of Oncology in South China, Collaborative Innovation Centre of Cancer Medicine, Guangdong Key Laboratory of Nasopharyngeal Carcinoma Diagnosis and Therapy, Sun Yat-sen University Cancer Center, Guangzhou, 510060, P.R. China.

**Keywords:** Protein-based signature, liquid biopsy, distant metastasis, Nasopharyngeal carcinoma, induction chemotherapy benefit

## Abstract

**Rationale:** Currently, for locoregionally advanced nasopharyngeal carcinoma (LA-NPC), there is no effective blood-based method to predict distant metastasis. We aimed to detect plasma protein profiles to identify biomarkers that could distinguish patients with NPC who are at high risk of posttreatment distant metastasis.

**Methods:** A high-throughput antibody array was initially applied to detect 1000 proteins in pretreatment plasma from 16 matched LA-NPC patients with or without distant metastasis after radical treatment. Differentially expressed proteins were further examined using a low-throughput array to construct a plasma protein-based signature for distant metastasis (PSDM) in a cohort of 226 patients.

**Results:** Fifty circulating proteins were differentially expressed between metastatic and non-metastatic patients and 18 were proven to be strongly correlated with distant metastasis-free survival (DMFS) in NPC. A PSDM signature consisting of five proteins (SLAMF5, ESM-1, MMP-8, INSR, and Serpin A5) was established to assign patients with NPC into a high-risk group and a low-risk group. Patients in the high-risk group had shorter DMFS (*P* < 0.001), disease-free survival (DFS) (*P* < 0.001) and overall survival (OS) (*P* < 0.001). Moreover, the PSDM performed better than N stage and Epstein-Barr virus (EBV) DNA load at effectively identifying patients with NPC at high risk of metastasis. For patients in the high-risk group, induction chemotherapy significantly improved DMFS, DFS, and OS.

**Conclusions:** The PSDM could be a useful liquid biopsy tool to effectively predict distant metastasis and the benefit of induction chemotherapy in patients with LA-NPC.

## Introduction

Nasopharyngeal carcinoma (NPC), an aggressive head and neck cancer, is highly prevalent in east and southeast Asia, especially southern China 1. More than 70% of patients with NPC present with advanced diseases at first diagnosis 2. Locoregional control has been greatly improved using radical chemoradiotherapy in patients with locoregionally advanced NPC (LA-NPC); however, distant metastasis has become the main reason for treatment failure 3, 4. Currently, tumor-node-metastasis (TNM) staging and the plasma Epstein-Barr virus (EBV) DNA load are used to predict metastasis in patients with NPC 5-7. However, various outcomes have been observed in NPC patients with the same TNM stage or similar EBV DNA load after receiving similar treatment, which suggests that these methods to predict prognosis and patient risk are not particularly accurate. Therefore, there is an urgent need to identify novel biomarkers to improve the risk assessment of metastasis in patients with NPC.

Liquid biopsy is a convenient and non-invasive sampling method that can dynamically reflect the changes of circulating components related to tumor diagnosis and prognosis 8. Metastasis is established in a multi-step manner dependent on interactions between cancer cells and the host environment 9, 10, in which the levels and composition of circulating proteins can be significantly altered 11, even before the actual distant relapse lesion is detectable by imaging. Proteins in circulation can reflect signals not only from the primary tumor, but also from potential micrometastatic diseases, as well as any inflammatory or immune response of the host, thereby representing the comprehensive risk for metastasis. Recently, advances in high-throughput technologies have led to the identification of differentially expressed proteins in the blood of patients with cancers, and collections of circulating proteins have been identified as novel cancer biomarkers 12, 13. Studies indicate that certain protein patterns might be helpful in prognostic prediction by comparing blood proteins from patients with NPC and healthy individuals 14, 15. However, the differences in pretreatment protein profiles that are useful to identify metastatic risks in patients with NPC who develop metastasis after treatment, compared with patients who do not, remain unexplored.

In the current study, we aimed to investigate whether circulating proteins could be used to determine the metastasis risk in patients with LA-NPC. Using antibody arrays, we compared the differential pretreatment plasma protein profiles between LA-NPC patients with or without posttreatment metastasis. Then, we developed a plasma protein-based signature for distant metastasis (PSDM) consisting of five markers, which showed improved efficacy compared with that of single proteins, N stage, and EBV DNA load to predict metastasis. Moreover, the PSDM classifier could identify patients who might benefit from induction chemotherapy.

## Methods

### Patient population and plasma collection

We collected 228 plasma samples from pretreatment patients with non-metastatic stage III-IVa LA-NPC at Sun Yat-sen University Cancer Center (Guangzhou, China) between July 2010 and December 2016. At the time of diagnosis, the blood was collected and centrifuged under anticoagulant conditions at 2330 × *g* for 10 minutes at 4 °C. The plasma was stored at -80 °C until analysis. No patients had received any anti-tumor therapy before blood sampling.

The 8th edition of the American Joint Committee on Cancer (AJCC) Staging Manual was used to pathologically diagnose and restage all the patients 16, and none of them had infectious diseases or autoimmune diseases at the time of diagnosis. All patients received radical radiotherapy (RT), including two-dimensional radiotherapy (2D-RT), three-dimensional conformal radiotherapy (3D-CRT), or intensity-modulated radiation therapy (IMRT). The primary tumor received a cumulative radiation dose of 66 Gy or greater and the involved neck area received 60-70 Gy. All bilateral cervical lymphatics and potential local infiltration sites received 50 Gy or greater. All patients were treated with 30-35 fractions with five daily fractions per week for 6-7 weeks. Platinum-based chemotherapy was administered to the patients, including induction chemotherapy (IC) and concurrent chemoradiotherapy (CCRT). IC consisted of cisplatin with 5-fluorouracil, taxanes, or both every 3 weeks for two or three cycles. CCRT consisted of 80-100 mg/m^2^ cisplatin on weeks 1,4, and 7 of radiotherapy or 35-40 mg/m^2^ cisplatin weekly 1.

For the induction chemotherapy benefit analysis, propensity score matching was adopted to adjust for patient selection bias and to generate two clinicopathologically matched groups with or without Taxane-Platinum-5FU (TPF) IC. The propensity score was calculated based on host and tumor factors, including age, sex, T stage, N stage, EBV DNA levels, and radiotherapy methods using the caliper algorithm with 0.05 by “MatchIT” packages in the R software (version 3.6.1) 17. All plasma EBV DNA levels were measured in the laboratory in the Department of Molecular Diagnosis at Sun Yat-sen University Cancer Center. Our laboratory has received accreditation from Stanford University, which led the international collaboration to harmonize the quantitative plasma EBV DNA assay and to undertake qualitative EBV DNA testing.

This study was approved by the Institutional Ethical Review Boards of the Sun Yat-Sen University Cancer Center, and the written informed consent was obtained from all patients. The results of the present study are reported according to the Reporting Recommendations for Tumor Marker Prognostic Studies (REMARK) criteria 18.

### Quantification of proteins by antibody array

High-throughput plasma protein profiles were acquired at the non-metastatic phase of eight patients with LA-NPC, who developed posttreatment metastasis during follow-up, and eight matched patients with LA-NPC, who did not, using a Quantibody Human Kiloplex Proteomics Array (Raybiotech, Inc, Norcross, GA, USA) according to the manufacturer's instructions. A total of 1000 proteins were detected semiquantitatively, including those related to immunity or inflammation (the most representative proteins were cytokines, such as those in the interleukin family, interferons, and chemokines), angiogenesis, cell growth, apoptosis, and signal transduction.

Each protein was analyzed in quadruplicate together with positive controls in the array, using a sandwich assay detection technique 19. The signal values were visualized through the addition of the streptavidin-conjugated Cy3 equivalent dye, using an Axon GenePix scanner (GenePix version 5.0, Axon Instruments, Union City, CA, USA), and then extracted for further analysis using Raybiotech Analyzer software. Raw signals were normalized by calculating a scaling factor for each array (normalized average of positive controls of all arrays/ average of positive controls in each array). For semi-quantitative high-throughput analysis, protein levels are given in terms of signal values. The normalized signal intensity values were log2‑transformed and differentially expressed proteins between patients with LA-NPC with and without metastasis were characterized according to the following criteria: Raw average signals of either group ≥ 300, fold change ≥ 1.5 or ≤ 0.67, and a t-test *P* value < 0.05.

Next, the differentially expressed proteins without cross-reaction were measured using a low-throughput customized quantitative Quantibody Human Array in a larger population of 226 patients with LA-NPC. The standard proteins were assayed simultaneously in each array together with the samples using a sandwich-based procedure to generate standard curves. By comparing normalized signals from unknown samples to the standard curve, the protein concentration in the samples was determined, and then log2-transformed before being subjected to subsequent analyses. We filtered out the proteins with concentrations below the lower limit of detection of the quantitative array in more than 50% of samples.

### Construction of the PSDM

To construct the PSDM, we first performed univariate Cox analysis to select proteins that correlated with distant metastasis-free survival (DMFS). Given the aim to identify candidate proteins that are highly related to metastasis and that the strictness of multiple test correction might rule out some of these potential markers, we included protein candidates with unadjusted *P* values less than 0.05 in the model construction. We then chose overexpressed proteins and adopted a LASSO Cox regression method to select candidates for the PSDM using the “glmnet” package in R software. The LASSO algorithm is a popular machine learning method, which is broadly applied to the Cox proportional hazard regression models for survival analysis 20-22. To determine the optimal values of λ, we used 10-time cross-validations with the 1-SE criteria. Then we selected proteins with nonzero coefficients based on the optimal values of λ, to construct the prediction model. The coefficients weighted by the Cox model were used to calculate the PSDM score (as detailed in the Supplementary Methods). The optimal PSDM cutoff value was selected with the “survivalROC” package based on the time-dependent ROC curve 23. The threshold that had the maximum sum of sensitivity and specificity for 5-year DMFS was used to separate patients into low-risk and high-risk groups.

### Statistical analysis

Our primary endpoint was DMFS, with disease-free survival (DFS) and overall survival (OS) as the secondary endpoints. DMFS was calculated from the first day of treatment to the date of first distant relapse or death from any cause, whichever occurred first. DFS was calculated from the first day of treatment to the date of the first relapse at any site or death from any cause, whichever occurred first. OS was calculated from the first day of treatment to the date of death from any cause. The Kaplan-Meier method and the log-rank test were used to estimate DMFS, DFS, and OS. Cox regression analyses were used to calculate the hazard ratios (HRs).

We assessed the differentially expressed proteins between the metastatic and nonmetastatic group with Student's t-test. The χ^2^ test or Fisher's exact test were used to compare categorical variables. We used univariate Cox analysis to analyze the correlations between the clinical outcomes and factors such as the PSDM, age, sex, T stage, N stage, and EBV DNA load. The factors that correlated significantly with the outcomes were further tested with multivariate Cox regression analysis. The predictive efficiency with regard to metastasis was compared between the PSDM and single proteins, as well as between the PSDM and clinical factors, using time-dependent receiver operating characteristic (ROC) curves and time-dependent area under the curve (AUC) analysis. We also performed a subgroup analysis with the Kaplan-Meier method and log-rank test to evaluate whether the PSDM could distinguish patients with better outcomes from those with worse outcomes in the groups with different stages and EBV DNA levels. All statistical tests were two-sided and differences were deemed significant at a *P* value < 0.05. R software (version 3.6.1) was used to perform the statistical analyses.

## Results

### Patient characteristics

We included 228 pretreatment patients with non-metastatic LA-NPC in this study. For the high-throughput profiles, eight patients with posttreatment metastasis and eight patients without posttreatment metastasis were strictly matched by age, sex, TNM stage, EBV DNA levels, and treatment methods to rule out the potential influence of those factors on metastasis (Table S1). We then included 226 pretreatment patients with non-metastatic LA-NPC for further study, among whom 14 patients were also enrolled in the profile analysis. Table 1 shows their clinical characteristics. The male to female ratio was 3.35:1 (174 males and 52 females). Among all the patients, 132 (58.4%) had stage III disease, and 94 (41.6%) had stage IVa disease. All patients underwent radical radiotherapy (2D-RT 1.8%, 3D-CRT or IMRT 98.2%), 211 patients (93.4%) received CCRT ± IC, and 15 patients (6.6%) received RT ± IC. The median follow-up was 87.5 months (interquartile range (IQR) 76.9-96.9). The median time interval between blood sampling and commencement of treatment was 12 days (IQR 6-20 days), and the time interval was not associated with survival (Table S2). Of the 226 patients, 41 (18.1%) developed distant metastasis, 65 (28.8%) suffered disease progression, and 42 (18.6%) died.

### Expression pattern and prognostic value of plasma proteins

**Figure 1:**
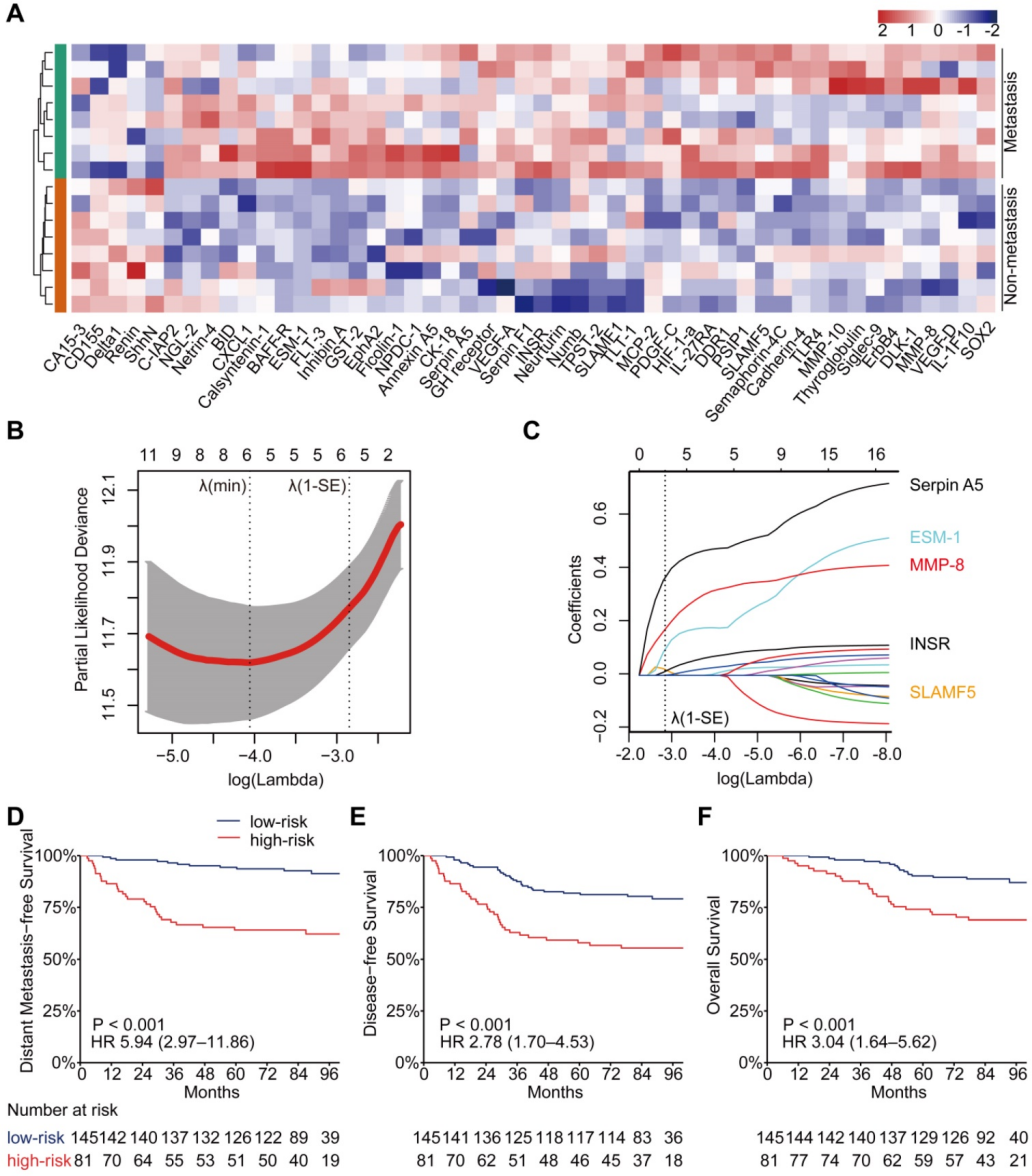
** Identification of differentially expressed proteins, construction of the PSDM, and Kaplan-Meier curves for clinical outcomes according to the PSDM.** (**A**) Heatmap of expression profiling of differentially expressed proteins between metastatic and nonmetastatic LA-NPC patients. Both the row and column were unsupervised and clustered with the hierarchical clustering method. (**B**) Ten-time cross-validations to tune the parameter selection in the LASSO model. The two dotted vertical lines are drawn at the optimal values by minimum criteria (left) and 1-SE criteria (right). (**C**) LASSO coefficient profiles of the candidate proteins for PSDM construction. A vertical line is drawn at the optimal value by 1-SE criteria and results in five non-zero coefficients: SLAMF5, ESM-1, MMP-8, INSR, and Serpin A5. (**D**) Kaplan-Meier curves for distant metastasis-free survival according to the PSDM. (**E**) Kaplan-Meier curves for disease-free survival according to the PSDM. (**F**) Kaplan-Meier curves for overall survival according to the PSDM. Abbreviations: LA-NPC, locoregionally advanced nasopharyngeal carcinoma; PSDM, protein-based signature for distant metastasis. HR, hazard ratio; and CI, confidence interval.

In the high-throughput proteomic array analysis, 50 proteins were found to be differentially expressed among 1000 proteins detected between 16 matched NPC patients with or without metastasis (Figure S1A, *P* < 0.05). These proteins showed strong classification properties in distinguishing the two groups (Figure 1A). Next, the 50 proteins were tested in a larger sample of 226 patients with LA-NPC using low-throughput customized antibody arrays. Eight proteins with concentrations below the lower limit of quantitative array detection in more than half of the patients were excluded from further study. Then, we compared the plasma protein levels between the 41 patients who developed distant metastases after radical treatment and 185 patients who did not. The results showed that 38 of the 42 (90.5%) proteins that were measured in both the high- and low-throughput arrays showed consistent trends associated with metastasis (both upregulated or both downregulated, Table S3). Among them, eighteen proteins were validated to be significantly differentially expressed between the patients with or without posttreatment distant metastasis (Table S3, Figure S1B, *P* < 0.05).

DMFS was the primary endpoint in the current study; therefore, we adopted univariate Cox regression analysis to identify DMFS-related proteins candidates for PSDM signature construction. The results showed that 18 candidates correlated significantly with DMFS (Table S4, *P* < 0.05). Seventeen of them were upregulated and one was downregulated in patients with distant metastasis after treatment. Statistically, 15 of the 18 prognostic proteins were significantly and differentially expressed (Table S5, *P* < 0.05). In addition, we also discovered that 13 proteins associated significantly with DFS, and 11 proteins correlated significantly with OS (Table S6-S7, *P* < 0.05). The cutoff value of each protein concentration that had the maximum sum of sensitivity and specificity to predict 5-year DMFS, based on the time-dependent ROC curve, was used to separate patients into low and high expression groups. The Kaplan-Meier curves of DMFS, DFS, and OS according to the low and high expression of these proteins in patients with NPC are provided in Figure S2-S4.

### PSDM construction and its association with prognosis

We selected the 17 upregulated proteins that were significantly associated with DMFS to construct a PSDM. We adopted a LASSO Cox regression model and identified five proteins that were strongly predictive of DMFS, namely, signaling lymphocytic activation molecule family member 5 (SLAMF5), endothelial cell-specific molecule 1 (ESM-1), insulin receptor (INSR), matrix metalloproteinase 8 (MMP-8) and serpin family A member 5 (Serpin A5) (Figure 1B-C). Then, a risk score for PSDM was calculated using a formula that included the five proteins weighted by their regression coefficients in a penalized Cox model as follows: Risk score = 0.0208 × the concentration of SLAMF5 + 0.1039 × the concentration of ESM‑1 + 0.1761 × the concentration of MMP-8 + 0.0161 × the concentration of INSR + 0.3738 × the concentration of Serpin A5.

**Figure 2:**
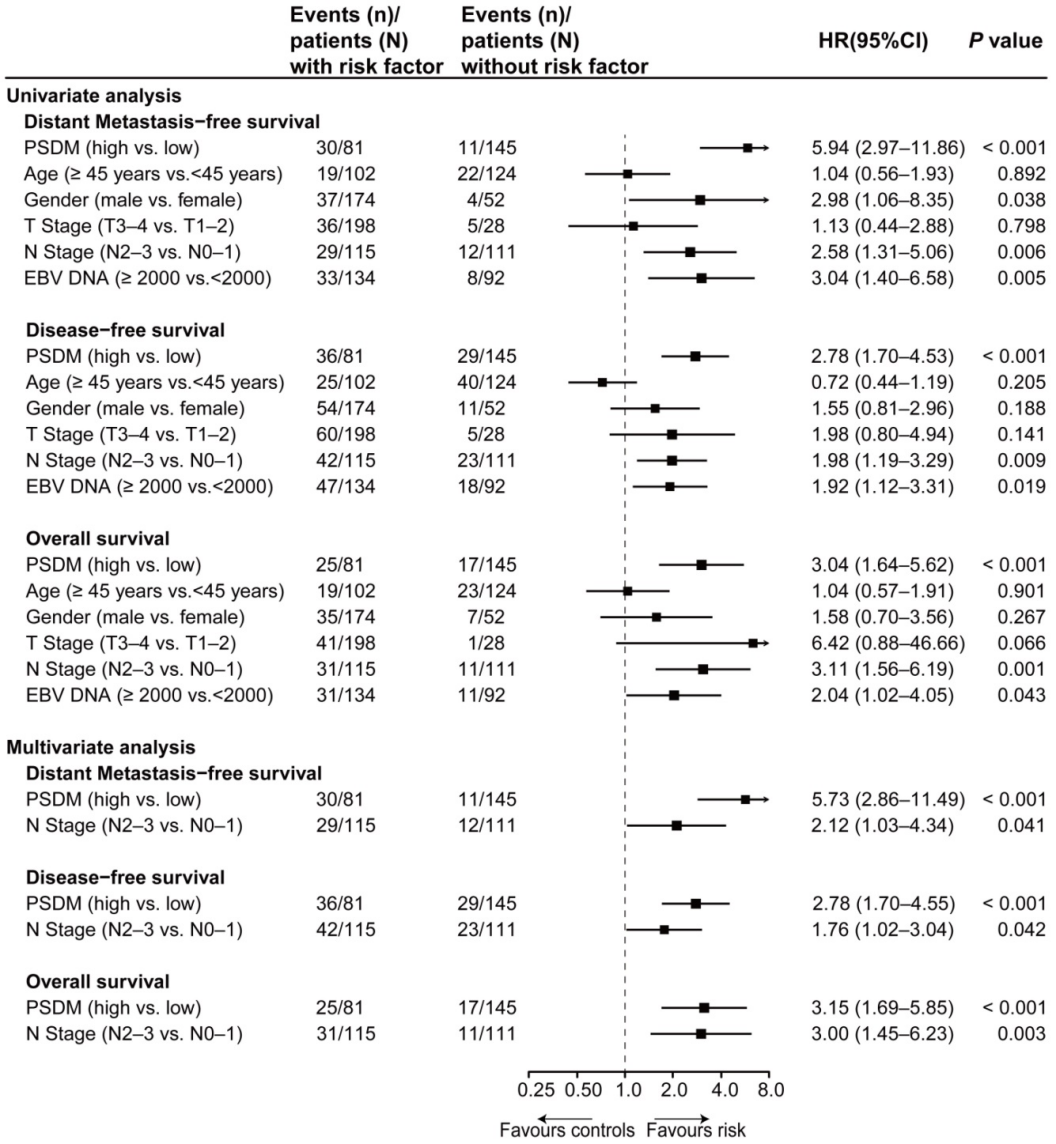
** Univariate and multivariate associations of the PSDM and clinicopathological characteristics with clinical outcomes.** Abbreviations: PSDM, protein-based signature for distant metastasis. HR, hazard ratio; and CI, confidence interval.

Using the optimal cutoff value (7.6030) for the PSDM, we classified 145 patients into the low-risk group and 81 patients into the high-risk group. Each of the five proteins in the PSDM signature was significantly upregulated in the high-risk group (Table S8, *P* < 0.001). Patients in the high-risk group had a shorter 5-year DMFS (64.2% vs. 94.4%, HR 5.94, 95% confidence interval (CI) 2.97-11.86, *P* < 0.001), 5-year DFS (58.0% vs. 81.9%, HR 2.78, 95% CI 1.70-4.53, *P* < 0.001) and 5-year OS (74.1% vs. 90.2%, HR 3.04, 95% CI 1.64-5.62, *P* < 0.001) than the low-risk group (Figure 1D-F). We performed univariate Cox regression to assess the associations between patient clinical outcomes and the PSDM, as well as the clinical outcomes and clinicopathological factors. A cutoff level of 2,000 copies/mL was used to separate patients into low and high pretreatment EBV DNA groups 6. The PSDM, N stage and EBV DNA levels were associated significantly with DMFS, DFS and OS (*P* < 0.05, Figure 2). Furthermore, when patients were stratified by the TNM stage, N stage, or T stage respectively, the PSDM was still associated with prognosis in all subgroups of patients, except the T1-2 subgroup (Figure S5-S7). Multivariate analyses showed that the PSDM remained a powerful and independent prognostic factor for any clinical outcome after adjustment for clinical variables (DMFS: HR 5.73, 95% CI 2.86-11.49, *P* < 0.001; DFS: HR 2.78, 95% CI 1.70-4.55, *P* < 0.001; OS: HR 3.15, 95% CI 1.69-5.85, *P* < 0.001) (Figure 2).

### Comparing the efficacy of PSDM with individual protein and clinical factors

**Figure 3:**
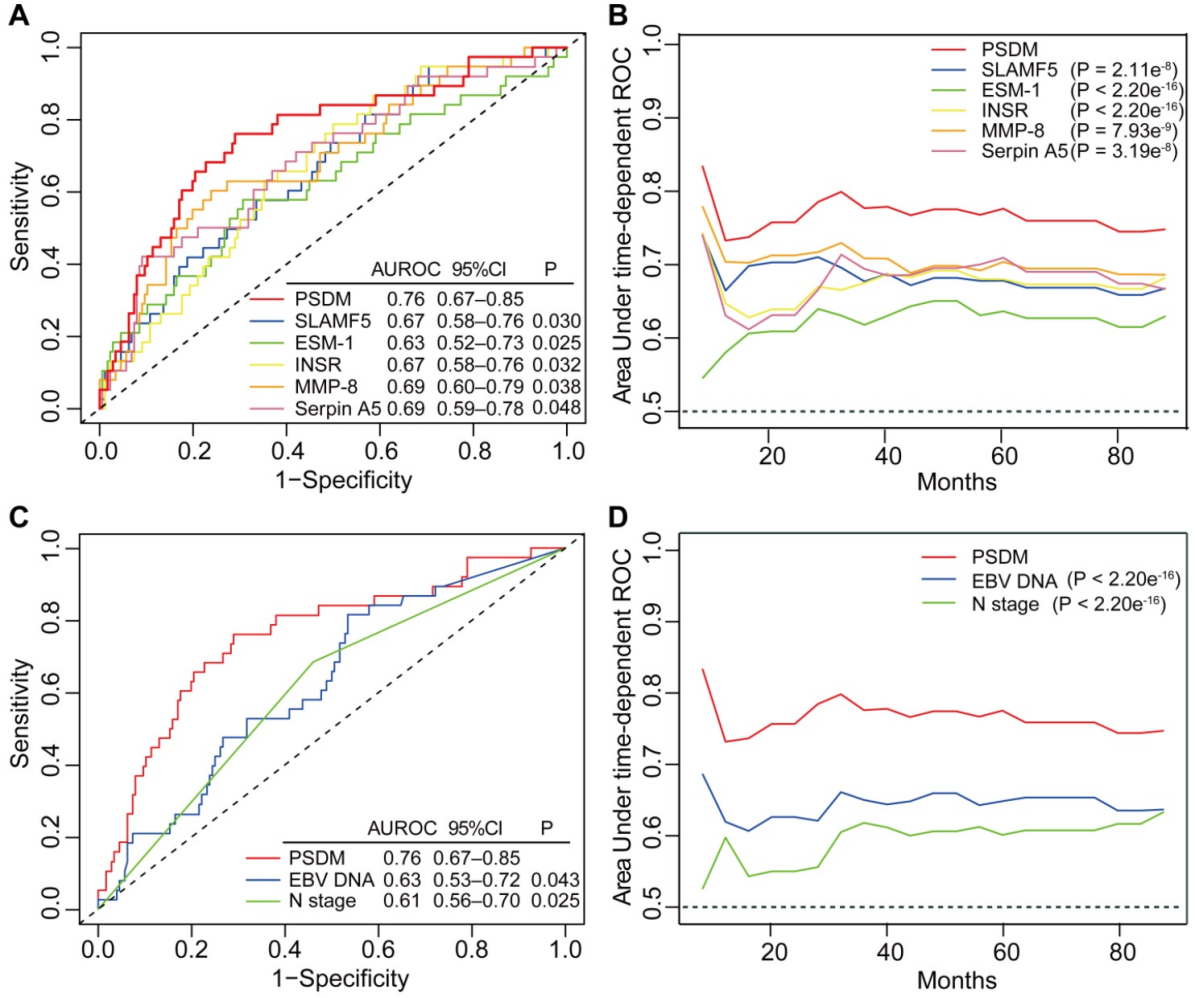
** Time-dependent ROC and AUC analysis of the PSDM, individual proteins, N stage and EBV DNA load for the prediction of metastasis.** (**A**) Time-dependent ROC analysis compared the PSDM and individual proteins for 5-year DMFS. (**B**) Time-dependent AUC analysis of DMFS compared the PSDM and individual proteins in a continuous period. (**C**) Time-dependent ROC analysis compared the PSDM, N stage and EBV DNA load for 5-year DMFS. (**D**) Time-dependent AUC analysis of DMFS compared the PSDM, N stage and EBV DNA load in a continuous period. Abbreviations: PSDM, protein-based signature for distant metastasis. HR, hazard ratio; and CI, confidence interval; EBV, Epstein-Barr virus.

To further evaluate the predictive performance of the PSDM for metastasis, we first determined whether the PSDM outperformed each protein. The results of time-dependent ROC analysis showed that the PSDM displayed better performance than each protein at predicting 5-year DMFS (*P* < 0.05, Figure 3A). Moreover, the PSDM outperformed individual protein consistently and stably over a long period, which was confirmed by time-dependent AUC analysis (*P* < 0.05, Figure 3B). N stage and pretreatment EBV DNA load are significantly associated with survival of patients with NPC in univariate Cox analysis and are often considered as prognostic indicators in NPC; therefore, we evaluated the prognostic performance of N stage and pretreatment EBV DNA load versus that of the PSDM. The PSDM displayed better efficacy than N stage and EBV DNA load with regard to distinguishing patients at high risk of metastasis (*P* < 0.05, Figure 3C-D).

### Stratification analysis with EBV DNA levels

**Figure 4:**
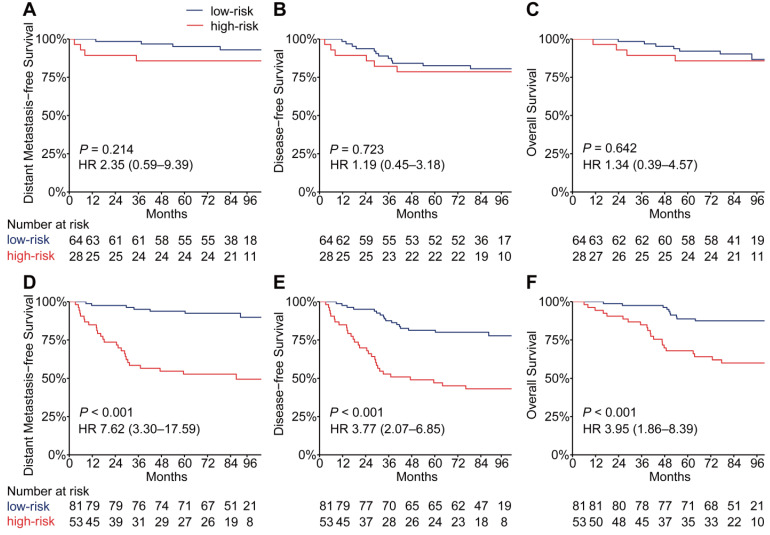
** Kaplan-Meier curves for distant metastasis-free survival, disease-free survival and overall survival of patients grouped by their EBV DNA level and then stratified according to the PSDM.** Plots show (**A**) distant metastasis-free survival, (**B**) disease-free survival and (**C**) overall survival in the EBV DNA level < 2000 copies/mL subgroup and (**D**) distant metastasis-free survival, (**E**) disease-free survival and (**F**) overall survival in the EBV DNA level ≥ 2000 copies/mL subgroup. Abbreviations: PSDM, protein-based signature for distant metastasis. HR, hazard ratio; and CI, confidence interval; EBV, Epstein-Barr virus.

EBV infection correlates strongly with head and neck squamous cell carcinoma, especially NPC 24. To evaluate the performance of the PSDM in patients with high and low EBV DNA burden at a cut-off level of 2,000 copies/mL 6, we conducted a subgroup analysis. In patients with high EBV DNA levels, the PSDM maintained outstanding performance in differentiating between patients with significantly different outcomes (DMFS: HR 7.62, 95% CI 3.30-17.59, *P* < 0.001; DFS: HR 3.77, 95% CI 2.07-6.85, *P* < 0.001; OS: HR 3.95, 95% CI 1.86-8.39, *P* < 0.001). However, in patients with EBV DNA burden < 2000 copies/ml, the Kaplan-Meier curves showed no significant association between the PSDM and any clinical outcome (Figure 4). The 5-year DMFS, DFS, and OS rates and the number of events in each group are listed in Table S9 and Table S10, respectively. Furthermore, we also found that in patients with high EBV DNA levels, the PSDM risk scores were significantly higher than in patients with low EBV DNA levels (*P* = 0.046, Figure S8).

### PSDM performance in predicting the benefit of TPF induction chemotherapy

**Figure 5:**
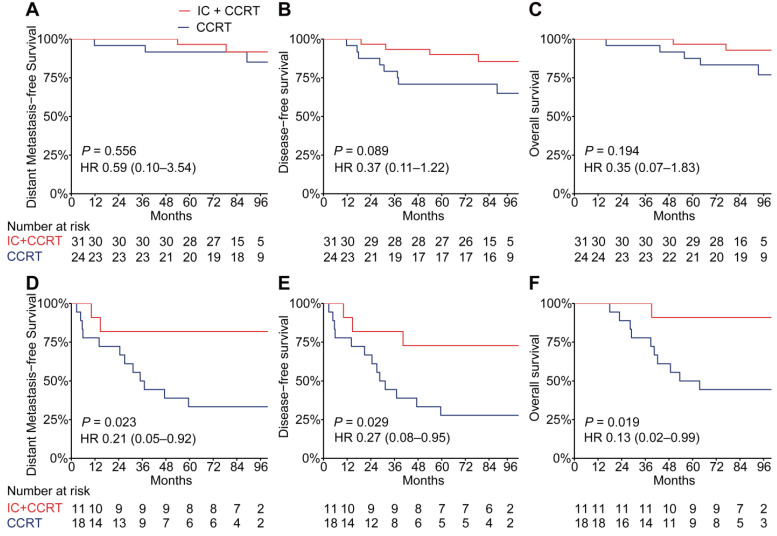
** Kaplan-Meier curves for distant metastasis-free survival, disease-free survival and overall survival of patients with TFP IC plus CCRT versus patients with CCRT alone stratified according to the PSDM.** Plots show (**A**) distant metastasis-free survival, (**B**) disease-free survival and (**C**) overall survival in patients with PSDM low risk scores and (**D**) distant metastasis-free survival, (**E**) disease-free survival and (**F**) overall survival in patients with PSDM high risk scores. Abbreviations: TPF, Taxane-Platinum-5FU regimen; IC, induction chemotherapy; CCRT, concurrent chemoradiotherapy; PSDM, protein-based signature for distant metastasis. HR, hazard ratio; and CI, confidence interval.

The addition of TPF IC to cisplatin-based CCRT has been proven to significantly improve distant metastasis-free survival in LA-NPC 25-27; therefore, we analyzed whether the PSDM could also be used to predict the efficacy of TPF IC. To adjust for patient selection bias, we used propensity score matching to choose 42 patients with LA-NPC who underwent TFP IC plus cisplatin-based CCRT and 42 matched patients with LA-NPC who underwent CCRT alone (cisplatin every 3 weeks ≥ 2 cycles) (Table S11). When the Kaplan-Meier survival analysis was stratified by the PSDM, compared with CCRT alone, treatment with TFP IC plus CCRT show a significant association with improved DMFS (HR 0.21, 95% CI 0.05-0.92, *P* = 0.023), DFS (HR 0.27, 95% CI 0.08-0.95, *P* = 0.029) and OS (HR 0.13, 95% CI 0.02-0.99, *P* = 0.019) in patients in the high-risk group but not in patients in the low-risk group (Figure 5).

## Discussion

In the present study, five circulating protein markers that could predict metastasis effectively in patients with LA-NPC were used to develop a novel prognostic model. Patients who are at high risk of distant metastasis, and might benefit from TPF IC, could be identified using the PDSM score. Furthermore, the PSDM performed better than single proteins, N stage, and EBV DNA levels with regard to predicting metastasis in patients with LA-NPC. To the best of our knowledge, this is the first study to apply a high-throughput method to evaluate plasma proteins profile in LA-NPC patients with and without posttreatment metastasis and to construct a protein-based metastasis signature for NPC.

Distant metastasis is the main reason for treatment failure in patients with NPC 3, 4. Currently, the most widely used tools, TNM stage and pretreatment EBV DNA, show poor performance to predict distant metastasis in NPC, which highlights the need to explore novel biomarkers with better efficacy. Advances in proteomic technology have facilitated the generation of multiprotein profiles, which can be used for risk stratification and therapeutic monitoring in cancer. Given the relative ease of blood sampling, protein signature-based liquid biopsy could be a promising choice for biomarker applications. Although a few low-throughput studies have identified differentially expressed proteins between patients with NPC and healthy individuals 14, 15, differences in blood protein profiles between NPC patients with different prognoses have been largely unexplored. Here, we compared up to 1000 proteins between matched plasma samples collected from patients with or without metastasis and identified a panel of five proteins with a strong ability to assess the risk of distant metastasis in patients with LA-NPC. Moreover, this PSDM classifier remained an independent and robust prognostic factor after adjustment for clinical characteristics.

The five proteins that compose the PSDM have been reported to be associated with pathophysiological processes in cancers, especially in processes related to metastasis 28-35. SLAMF5, a member of the SLAM family of cell-surface immunoreceptors, is believed to modulate the activation and differentiation of immune cells and to regulate the survival of chronic lymphocytic leukemia cells 28, 29. ESM-1 was found to be involved in angiogenesis and is highly expressed on tumor vessels in bladder and gastric cancers 30, 31. MMP-8 is believed to promote the invasion of cancer cells and to serve as a prognostic marker in colorectal cancer 32, 33. It is thought that INSR is related to increased cell proliferation and metastatic potential 34, while Serpin A5 has been found to play dual roles in cancer, inhibiting the growth of tumors but promoting tumor metastasis 35. These findings also indicated that the five proteins of the PSDM signature were probably not specific to NPC, which could be furthered demonstrated by the Human Protein Atlas database (https://www.proteinatlas.org/) 36, 37, a database showing the expression levels of protein across tissues in normal and pathological conditions. Moreover, metastasis is a systemic process. The dysregulated circulating proteins would originate from the primary tumor, micrometastatic disease, or could be a reflection of the host state, which makes the PSDM a comprehensive signature for metastatic risk assessment. Therefore, the five proteins of the PSDM signature probably reflected the pathological process related to metastasis rather than a specific kind of cancer, showing promising potential as cancer biomarkers for NPC metastasis prediction.

We also performed a stratification analysis to determine the prognostic value of the PSDM in patients with different EBV DNA loads. In the subgroup with high EBV DNA levels, our PSDM maintained outstanding efficacy to discriminate among patients with distinctly different outcomes; however, this was not true in the low EBV DNA subgroup. It has been noted that the EBV DNA load may reflect the potential metastasis risk in patients with NPC 5-7. In other words, patients with low EBV DNA burdens may have a lower metastatic risk. Our analysis indicated that the number of metastatic events and the PSDM risk scores were lower in the low EBV DNA subgroup, which was in accordance with the findings of previous studies. Taken together, the PSDM was unable to effectively discriminate high-risk patients from low-risk patients due to the overall lower risk of patients in the low EBV DNA subgroup. Thus, we believe that the PSDM model would be better applied in patients with high EBV DNA levels as it allows for more accurate classification of patients at high risk.

Accumulating evidence shows that induction chemotherapy could provide a survival benefit by improving distant control in NPC 27, probably because of its systemic tumor-suppressing effect, which eradicates micrometastases early. Our previous phase 3 study showed that TPF IC added to CCRT resulted in a significant decrease in distant metastasis and prolonged survival in patients with LA-NPC 25, 26. However, the responses to TPF IC remain heterogeneous. In this study, we showed that the addition of TFP IC to CCRT provided a survival benefit in patients with high PSDM risk scores, but not in patients with low risk scores. Therefore, the PSDM could also serve as a tool to predict the efficacy of TFP IC. Moreover, variations in EBV DNA clearance kinetics have been reported to effectively cluster patients into different IC response phenotypes 38. Taken together, it might be complementary to combine our PSDM and EBV DNA tracking to predict the benefit of IC in the future. On the one hand, patients who were defined as non-beneficiaries for IC by the PSDM, might undergo CCRT directly to avoid additional adverse effects of IC. On the other hand, patients who were identified as beneficiaries for IC might receive IC, and EBV DNA tracking could provide useful information for risk-adapted treatment de-intensification or intensification during IC.

Our study was limited by the lack of patients with early-stage disease (TNM stage I-II); therefore, the PSDM might not be suitable or may need to be modified for early-stage patients. In addition, we also acknowledge that this study was limited by its retrospective and single-center nature. The current marker panel requires further validation by other methods, such as enzyme-linked immunosorbent assays (ELISA) and prospective evaluation in independent cohorts before its clinical application.

In conclusion, we developed a plasma protein-based signature consisting of five proteins that performed much better than clinical indicators to predict the distant metastasis of NPC. Moreover, we showed that the PSDM classifier could identify patients who might benefit from TPF IC. Therefore, the PSDM, which is easily detected in peripheral blood, might be a useful liquid biopsy tool to classify patients with different metastatic risks and could lead to more individualized treatment of LA-NPC.

## Supplementary Material

Supplementary figures and tables.

## Figures and Tables

**Table 1:** Patients' characteristics stratified according to the protein-based signature for distant metastasis (PSDM)

	All	Low-risk	High-risk	*P*
Total population	226 (100)	145 (64.2)	81 (35.8)	
**Age**				0.394
< 45 years	124 (54.9)	76 (52.4)	48 (59.3)	
≥ 45 years	102 (45.1)	69 (47.6)	33 (40.7)	
**Gender**				0.481
Male	174 (77.0)	109 (75.2)	65 (80.3)	
Female	52 (23.0)	36 (24.8)	16 (19.8)	
**T Stage**				0.551
T1	7 ( 3.1)	6 ( 4.1)	1 ( 1.2)	
T2	21 ( 9.3)	15 (10.3)	6 ( 7.4)	
T3	144 (63.7)	90 (62.1)	54 (66.7)	
T4	54 (23.9)	34 (23.5)	20 (24.7)	
**N Stage**				0.898
N0	13 ( 5.8)	8 ( 5.5)	5 ( 6.2)	
N1	98 (43.4)	63 (43.5)	35 (43.2)	
N2	67 (29.6)	45 (31.0)	22 (27.1)	
N3	48 (21.2)	29 (20.0)	19 (23.5)	
**TNM Stage**				0.611
III	132 (58.4)	87 (60.0)	45 (55.6)	
IVA	94 (41.6)	58 (40.0)	36 (44.4)	
**EBV DNA load (copies/mL)**				0.207
< 2000	92 (40.7)	64 (44.1)	28 (34.6)	
≥ 2000	134 (59.3)	81 (55.9)	53 (65.4)	
**Radiotherapy**				0.300
2D-RT	4 ( 1.8)	4 ( 2.8)	0 ( 0)	
3D-CRT or IMRT	222 (98.2)	141 (97.2)	81 (100)	
**Chemotherapy**				0.999
CCRT ± IC	211 (93.4)	135 (93.1)	76 (93.8)	
RT ± IC	15 ( 6.6)	10 ( 6.9)	5 ( 6.2)	
**Distant Metastasis**				< 0.001
Yes	41 (18.1)	11 ( 7.6)	30 (37.0)	
No	185 (81.9)	134 (92.4)	51 (63.0)	
**Disease progression**				< 0.001
Yes	65 (28.8)	29 (20.0)	36 (44.4)	
No	161 (71.2)	116 (80.0)	45 (55.6)	
**Death**				< 0.001
Yes	42 (18.6)	17 (11.7)	25 (30.9)	
No	184 (81.4)	128 (88.3)	56 (69.1)	

Abbreviations: TNM, tumor-node-metastasis.

## Abbreviations

LA-NPC: locoregionally advanced nasopharyngeal carcinoma
PSDM: protein-based signature for distant metastasis
DMFS: distant metastasis-free survival
DFS: disease-free survival
OS: overall survival
EBV: Epstein-Barr virus
TNM: tumor-node-metastasis stage
RT: radical radiotherapy
2D-RT: two-dimensional radiotherapy
3D-CRT: three-dimensional conformal radiotherapy
IMRT: intensity-modulated radiation therapy
IC: induction chemotherapy
CCRT: concurrent chemoradiotherapy
TPF: Taxane-Platinum-5FU regimen
ROC: receiver operating characteristic
AUC: area under the curve
IQR: interquartile range
HR: hazard ratio
CI: confidence interval
SLAMF5: signaling lymphocytic activation molecule family member 5
ESM-1: endothelial cell-specific molecule 1
INSR: insulin receptor
MMP-8: matrix metalloproteinase 8
Serpin A5: serpin family A member 5
ELISA: enzyme-linked immunosorbent assays
