# Supplementary material for: Plasma protein-based signature predicts distant metastasis and induction chemotherapy benefit in Nasopharyngeal Carcinoma
Source: Theranostics. 2020 Aug 1;10(21):9767–78. doi: 10.7150/thno.47882 (PMC7449924; doi:10.7150/thno.47882)
Supplement: Supplementary file 1 — Supplementary figures and tables. [file thnov10p9767s1.pdf]

# Supplementary Materials

## Plasma protein-based signature predicts distant metastasis and induction chemotherapy benefit in nasopharyngeal carcinoma

Yelin Liang, Junyan Li, Qian Li, Linglong Tang, Lei Chen, Yanping Mao, Qingmei He, Xiaojing Yang, Yuan Lei, Xiaohong Hong, Yin Zhao, Shiwei He, Ying Guo, Yaqin Wang, Panpan Zhang, Na Liu, Yingqin Li, Jun Ma.

## Supplementary Methods

## Supplementary Tables and Figures

**Table S1. Clinicopathological characteristics of 16 matched patients with post-treatment metastatic nasopharyngeal carcinoma (MNPC) and post-treatment non-metastatic nasopharyngeal carcinoma (NMNPC).**

**Table S2. Univariate Cox regression analysis to explore the impact of time interval on clinical outcomes.**

**Table S3. The result of differential expression analysis in high-throughput and low-throughput arrays.**

**Table S4. Univariate analysis of the 42 differently expressed proteins associated with distant metastasis-free survival.**

**Table S5. The results of Univariate Cox analysis and differential analysis of the 18 proteins significantly associated with distant metastasis-free survival.**

**Table S6. Univariate analysis of the 42 differently expressed proteins associated with disease-free survival.**

**Table S7. Univariate analysis of the 42 differently expressed proteins associated with overall survival.**

**Table S8. The concentration of the 5 proteins of PSDM signature in high and low metastatic risk group stratified by the PSDM risk score.**

**Table S9. Five-year distant metastasis-free survival, disease-free survival and overall survival estimates for different groups.**

**Table S10. The number of events for different groups.**

**Table S11. Clinicopathological characteristics of the 84 patients selected by propensity score matching in TPF IC benefit subgroup analysis.**

**Figure S1. Expression of plasma proteins related to metastasis in the high-throughput and low-throughput arrays.**

**Figure S2. Kaplan–Meier curves of distant metastasis-free survival (DMFS) according to the 18 proteins associated with DMFS.**

**Figure S3. Kaplan–Meier curves of disease-free survival (DFS) according to the 13 proteins associated with DFS.**

**Figure S4. Kaplan–Meier curves of overall survival (OS) according to the 11 proteins associated with OS.**

**Figure S5. Kaplan–Meier curves for patients with low risk and high risk according to the PSDM in stage III or stage IV subgroup.**

**Figure S6. Kaplan–Meier curves for patients with low risk and high risk according to the PSDM in T1–2 or T3–4 subgroup.**

**Figure S7. Kaplan–Meier curves for patients with low risk and high risk according to the PSDM in N0–1 or N2–3 subgroup.**

**Figure S8. The risk scores for PSDM in the low and high EBV DNA subgroups.**

# Supplementary Methods

## Constructing a protein-based signature for metastasis (PSDM) by LASSO cox regression analysis with ten-fold validation

The least absolute shrinkage and selection operator (LASSO) is a popular method for regression with high-dimensional predictors. It introduces a penalty parameter λ to shrink some regression coefficients to exactly zero. The penalty parameter λ, called the tuning parameter, controls the amount of shrinkage: the larger the value of λ, the fewer the number of predictors selected [1]. LASSO has been broadly applied to the Cox proportional hazard regression model for survival analysis to prevent overfitting [2–4]. We selected 17 DMFS-correlated plasma proteins with upregulated tendency and adopted a LASSO Cox regression model to achieve shrinkage and variable selection simultaneously. Ten-fold cross-validation was used to determine the optimal values of λ. In short, the 226 LA-NPC patients were randomly partitioned into 10 equal-sized subsamples. A series of different λ values for LASSO was generated by the “glmnet” package [2] in R software. For each λ, 9 subsamples were used as training data to generate a model, and the remaining 1 subsample was retained to validate the model. The partial likelihood deviance was calculated to evaluate the efficacy variation between the training and validation subsamples. The cross-validation process is then repeated 10 times, with each of the 10 subsamples used exactly once as the validation data. In this way, for each λ, the mean and estimated standard error of the partial likelihood deviances in ten times were calculated. We choose λ via 1-SE (standard error) criteria [3–4], i.e. the optimal λ is the largest value for which the partial likelihood deviance is within one SE of the smallest value of partial likelihood deviance (Figure 1B–C). Based on this λ value, we could obtain the variables whose beta coefficients were not zero, namely SLAMF5 (coefficient = 0.0208), ESM-1 (coefficient = 0.1039), MMP-8 (coefficient = 0.1761), INSR

(coefficient = 0.0161) and Serpin A5 (coefficient = 0.3738). Then we constructed the PSDM with these coefficients, so risk scores = 0.0208 × the concentration of SLAMF5 + 0.1039 × the concentration of ESM-1 + 0.1761 × the concentration of MMP-8 + 0.0161 × the concentration of INSR + 0.3738 × the concentration of Serpin A5.

**References:**


(1) Tibshirani R. Regression shrinkage and selection via the LASSO. J R Statist Soc Series B (Statistical Methodology). 1996.

(2) Tibshirani R. The lasso method for variable selection in the Cox model. Stat Med 1997; 16: 385-95

(3) Zhang JX, Song W, Chen ZH, Wei JH, Liao YJ, Lei J, et al. Prognostic and predictive value of a microRNA signature in stage II colon cancer: a microRNA expression analysis. Lancet Oncol. 2013; 14: 1295-306.

(4) Wei J, Feng Z, Cao Y, Zhao H, Chen Z, Liao B, et al. Predictive value of single-nucleotide polymorphism signature for recurrence in localised renal cell carcinoma: a retrospective analysis and multicentre validation study. Lancet Oncology. 2019; 20: 591-600.

**Table S1. Clinicopathological characteristics of 16 matched patients with post-treatment metastatic nasopharyngeal carcinoma (MNPC) and post-treatment non-metastatic nasopharyngeal carcinoma (NMNPC).**

| | MNPC N(%) | NMNPC N(%) | *P* |
|---|---|---|---|
| **Age(Mean ±SD)** | 38.4 ± 13.1 | 39.1 ± 12.8 | |
| **Gender** | | | 0.99 |
| Male | 6 (75.0) | 6 (75.0) | |
| Female | 2 (25.0) | 2 (25.0) | |
| **T Stage** | | | 0.99 |
| T3 | 5 (62.5) | 5 (62.5) | |
| T4 | 3 (37.5) | 3 (37.5) | |
| **N Stage** | | | 0.99 |
| N1 | 5 (62.5) | 5 (62.5) | |
| N2 | 2 (25.0) | 2 (25.0) | |
| N3 | 1 (12.5) | 1 (12.5) | |
| **TNM Stage** | | | 0.99 |
| III | 4 (50.0) | 4 (50.0) | |
| IV | 4 (50.0) | 4 (50.0) | |
| **EBV DNA load (copies/mL)** | | | 0.99 |
| < 2000 | 1 (12.5) | 2 (25.0) | |
| ≥ 2000 | 7 (87.5) | 6 (75.0) | |
| **Radiotherapy** | | | 0.99 |
| 3D-CRT or IMRT | 8 (100.0) | 8 (100.0) | |
| **Chemotherapy** | | | 0.99 |
| CCRT ±IC | 8 (100.0) | 8 (100.0) | |

Abbreviations: TNM: tumor-node-metastasis; 3D-CRT: three-dimensional conformal radiotherapy; IMRT: intensity-modulated radiation therapy; CCRT: concomitant chemoradiotherapy; IC: induction chemotherapy.

**Table S2. Univariate Cox regression analysis to explore the impact of time interval on clinical outcomes.**

| | HR | 95%CI | *P* |
|---|---|---|---|
| **Distant metastasis-free survival** | 1.024 | (0.992–1.056) | 0.148 |
| **Disease-free survival** | 1.016 | (0.990–1.042) | 0.225 |
| **Overall survival** | 1.011 | (0.979–1.043) | 0.512 |

**Table S3. The result of differential expression analysis in high-throughput and low-throughput arrays.**

| Protein | High-throughput array | | Low-throughput array | |
|---|---|---|---|---|
| | log2(Foldchange) (Metastasis/non-metastasis) | *P* | log2(Foldchange) (Metastasis/non-metastasis) | *P* |
| Annexin A5 | 1.05 | 0.019 | 0.83 | 0.028 |
| BAFF-R | 0.82 | 0.007 | 0.50 | 0.054 |
| BID | 0.77 | 0.031 | 0.97 | 0.183 |
| CA 15-3† | -0.71 | 0.030 | 0.68 | 0.067 |
| Cadherin-4 | 1.01 | 0.010 | 0.37 | 0.176 |
| Calsyntenin-1 | 0.91 | 0.035 | 0.22 | 0.601 |
| CD155† | -4.64 | 0.014 | 0.64 | 0.080 |
| GST-2 | 0.95 | 0.045 | 0.83 | 0.191 |
| C-IAP2 | 1.21 | 0.011 | 0.44 | 0.177 |
| CK-18 | 1.02 | 0.048 | 2.02 | 0.010 |
| CXCL1 | 1.29 | 0.033 | 0.18 | 0.851 |
| Delta1† | -4.32 | 0.042 | 0.29 | 0.184 |
| DLK-1 | 0.85 | 0.025 | 0.69 | < 0.001 |
| EphA2 | 1.35 | 0.030 | 0.36 | 0.087 |
| ErbB4 | 1.07 | 0.040 | 0.42 | 0.110 |
| ESM-1 | 0.70 | 0.004 | 0.40 | 0.029 |
| Ficolin-1 | 0.77 | 0.038 | 0.49 | 0.038 |
| FLT-3 | 1.24 | 0.014 | 0.71 | 0.076 |
| GH receptor | 0.70 | 0.038 | 0.46 | 0.024 |
| IL-1F10 | 0.67 | 0.031 | 0.26 | 0.536 |
| IL-27RA | 1.12 | 0.002 | 0.48 | 0.010 |
| INSR | 0.94 | 0.014 | 1.44 | < 0.001 |
| PSIP1 | 0.84 | 0.031 | 0.84 | 0.085 |
| MCP-2 | 1.09 | 0.026 | 0.36 | 0.023 |
| MMP-8 | 0.70 | 0.004 | 0.65 | < 0.001 |
| Neurturin | 1.07 | 0.006 | 0.32 | 0.024 |
| NPDC-1 | 0.60 | 0.04 | 0.36 | 0.015 |
| PDGF-C | 0.70 | 0.013 | 1.02 | 0.004 |
| Renin | -1.00 | 0.047 | -0.13 | 0.515 |
| Semaphorin-4C | 1.07 | 0.045 | 1.21 | 0.095 |
| Serpin A5 | 0.70 | 0.044 | 0.29 | 0.002 |
| Serpin F1 | 0.69 | 0.045 | 0.28 | 0.086 |
| ShhN | -0.81 | 0.015 | -0.24 | 0.094 |
| Siglec-9 | 0.63 | 0.024 | 0.37 | 0.019 |
| SLAMF1 | 0.99 | 0.028 | 0.36 | 0.029 |

| | | | | |
|---|---|---|---|---|
| SLAMF5 | 0.76 | 0.013 | 0.69 | < 0.001 |
| SOX2† | 0.75 | 0.009 | -0.27 | 0.676 |
| Thyroglobulin | 0.69 | 0.037 | 0.87 | 0.003 |
| TLR4 | 1.08 | 0.029 | 0.46 | 0.232 |
| TPST-2 | 1.01 | 0.027 | 0.56 | 0.409 |
| VEGF-A | 0.71 | 0.018 | 0.51 | 0.073 |
| VEGF-D | 0.64 | 0.045 | 0.09 | 0.644 |

†The proteins that showed inconsistent trends in high- and low-throughput arrays.

Abbreviations: ShhN: Sonic Hedgehog N-Terminal.

**Table S4. Univariate analysis of the 42 differently expressed proteins associated with distant metastasis-free survival.**

| | DMFS | | |
|---|---|---|---|
| | HR | 95% CI | *P* |
| Annexin A5 | 1.20 | (1.04–1.38) | 0.012 |
| BAFF-R | 1.23 | (1.00–1.50) | 0.049 |
| BID | 1.04 | (0.97–1.11) | 0.299 |
| CA 15-3 | 1.11 | (0.97–1.28) | 0.115 |
| Cadherin-4 | 1.20 | (0.97–1.49) | 0.089 |
| Calsyntenin-1 | 1.09 | (0.89–1.34) | 0.414 |
| CD155 | 1.1 | (0.96–1.27) | 0.178 |
| SLAMF5 | 1.46 | (1.20–1.78) | <0.001 |
| GST-2 | 1.06 | (0.97–1.17) | 0.189 |
| C-IAP2 | 1.12 | (0.94–1.34) | 0.202 |
| CK-18 | 1.11 | (1.03–1.19) | 0.009 |
| Delta1 | 1.08 | (0.90–1.30) | 0.416 |
| ESM-1 | 1.64 | (1.22–2.20) | 0.001 |
| EphA2 | 1.26 | (0.99–1.60) | 0.058 |
| ErbB4 | 1.20 | (0.95–1.51) | 0.127 |
| Ficolin-1 | 1.31 | (1.05–1.62) | 0.015 |
| FLT-3 | 1.16 | (1.00–1.35) | 0.051 |
| GH receptor | 1.30 | (0.99–1.70) | 0.062 |
| CXCL1 | 1.01 | (0.95–1.07) | 0.858 |
| IL-1F10 | 1.12 | (0.89–1.41) | 0.334 |
| IL-27RA | 1.61 | (1.17–2.20) | 0.003 |
| INSR | 1.33 | (1.13–1.57) | 0.001 |
| PSIP1 | 1.11 | (0.98–1.25) | 0.115 |
| MCP-2 | 1.35 | (0.99–1.85) | 0.058 |
| MMP-8 | 2.11 | (1.44–3.09) | <0.001 |
| Neurturin | 1.55 | (1.06–2.25) | 0.022 |
| NPDC-1 | 1.84 | (1.29–2.62) | 0.001 |
| PDGF-C | 1.26 | (1.03–1.53) | 0.026 |
| DLK-1 | 1.44 | (1.07–1.95) | 0.017 |
| Renin | 0.87 | (0.64–1.19) | 0.393 |
| Semaphorin-4C | 1.06 | (0.99–1.15) | 0.114 |
| Serpin A5 | 2.80 | (1.65–4.76) | <0.001 |
| Serpin F1 | 1.36 | (0.97–1.91) | 0.075 |
| ShhN | 0.62 | (0.39–0.97) | 0.037 |
| Siglec-9 | 1.54 | (1.06–2.25) | 0.024 |
| SLAMF1 | 1.32 | (1.00–1.75) | 0.050 |
| SOX2 | 0.98 | (0.90–1.07) | 0.687 |

| | | | |
|---|---|---|---|
| Thyroglobulin | 1.27 | (1.02–1.57) | 0.031 |
| TLR4 | 1.13 | (0.97–1.31) | 0.123 |
| TPST-2 | 1.04 | (0.95–1.13) | 0.405 |
| VEGF-A | 1.35 | (1.03–1.77) | 0.030 |
| VEGF-D | 1.07 | (0.77–1.48) | 0.685 |

Eight proteins whose concentrations were below LOD were excluded.

Abbreviations: DMFS: distant metastasis-free survival; ShhN: Sonic Hedgehog N-Terminal; LOD: the lower limit of detection.

**Table S5. The results of Univariate Cox analysis and differential analysis of the 18 proteins significantly associated with distant metastasis-free survival.**

| | Univariate analysis for DMFS | | | Student *t*-test | |
|---|---|---|---|---|---|
| | **HR** | **95% CI** | ***P*** | **log2(FoldChange)** | ***P*** |
| Annexin A5 | 1.20 | (1.04–1.38) | 0.012 | 0.83 | 0.028 |
| BAFF-R* | 1.23 | (1.00–1.50) | 0.049 | 0.50 | 0.054 |
| SLAMF5 | 1.46 | (1.20–1.78) | < 0.001 | 0.69 | < 0.001 |
| CK-18 | 1.11 | (1.03–1.19) | 0.009 | 2.02 | 0.010 |
| ESM-1 | 1.64 | (1.22–2.20) | 0.001 | 0.40 | 0.029 |
| Ficolin-1 | 1.31 | (1.05–1.62) | 0.015 | 0.49 | 0.038 |
| IL-27RA | 1.61 | (1.17–2.20) | 0.003 | 0.48 | 0.010 |
| INSR | 1.33 | (1.13–1.57) | 0.001 | 1.44 | < 0.001 |
| MMP-8 | 2.11 | (1.44–3.09) | < 0.001 | 0.65 | < 0.001 |
| Neurturin | 1.55 | (1.06–2.25) | 0.022 | 0.32 | 0.024 |
| NPDC-1 | 1.84 | (1.29–2.62) | 0.001 | 0.36 | 0.015 |
| PDGF-C | 1.26 | (1.03–1.53) | 0.026 | 1.02 | 0.004 |
| DLK-1 | 1.44 | (1.07–1.95) | 0.017 | 0.69 | < 0.001 |
| Serpin A5 | 2.80 | (1.65–4.76) | < 0.001 | 0.29 | 0.002 |
| ShhN* | 0.62 | (0.39–0.97) | 0.037 | -0.24 | 0.094 |
| Siglec-9 | 1.54 | (1.06–2.25) | 0.024 | 0.37 | 0.019 |
| Thyroglobulin | 1.27 | (1.02–1.57) | 0.031 | 0.87 | 0.003 |
| VEGF-A* | 1.35 | (1.03–1.77) | 0.030 | 0.51 | 0.073 |

*The 3 proteins that were significantly associated with distant metastasis-free survival in univariate Cox analysis but marginally significant in differential analysis with student t-test.

**Table S6. Univariate analysis of the 42 differently expressed proteins associated with disease-free survival.**

| | DFS | | |
|---|---|---|---|
| | HR | 95% CI | *P* |
| Annexin A5 | 1.20 | (1.07–1.34) | 0.002 |
| BAFF-R | 1.17 | (0.99–1.38) | 0.058 |
| BID | 1.02 | (0.97–1.08) | 0.431 |
| CA 15-3 | 0.98 | (0.89–1.09) | 0.751 |
| Cadherin-4 | 1.13 | (0.95–1.36) | 0.166 |
| Calsyntenin-1 | 1.09 | (0.93–1.29) | 0.294 |
| CD155 | 0.98 | (0.90–1.07) | 0.696 |
| SLAMF5 | 1.26 | (1.04–1.52) | 0.017 |
| GST-2 | 1.08 | (1.00–1.16) | 0.049 |
| C-IAP2 | 1.02 | (0.90–1.16) | 0.780 |
| CK-18 | 1.08 | (1.02–1.15) | 0.011 |
| Delta1 | 1.11 | (0.95–1.29) | 0.210 |
| ESM-1 | 1.50 | (1.16–1.95) | 0.002 |
| EphA2 | 1.22 | (1.01–1.48) | 0.043 |
| ErbB4 | 1.13 | (0.94–1.35) | 0.184 |
| Ficolin-1 | 1.27 | (1.07–1.52) | 0.007 |
| FLT-3 | 1.12 | (0.99–1.27) | 0.060 |
| GH receptor | 1.16 | (0.95–1.41) | 0.151 |
| CXCL1 | 0.99 | (0.94–1.03) | 0.535 |
| IL-1F10 | 1.09 | (0.91–1.30) | 0.365 |
| IL-27RA | 1.36 | (1.06–1.76) | 0.018 |
| INSR | 1.13 | (1.01–1.27) | 0.037 |
| PSIP1 | 1.09 | (0.99–1.19) | 0.086 |
| MCP-2 | 1.12 | (0.89–1.41) | 0.326 |
| MMP-8 | 1.54 | (1.16–2.03) | 0.003 |
| Neurturin | 1.41 | (1.05–1.91) | 0.023 |
| NPDC-1 | 1.62 | (1.19–2.20) | 0.002 |
| PDGF-C | 1.08 | (0.96–1.21) | 0.189 |
| DLK-1 | 1.07 | (0.90–1.26) | 0.445 |
| Renin | 0.85 | (0.67–1.09) | 0.214 |
| Semaphorin-4C | 1.03 | (0.97–1.09) | 0.309 |
| Serpin A5 | 1.99 | (1.26–3.14) | 0.003 |
| Serpin F1 | 1.13 | (0.85–1.50) | 0.392 |
| ShhN | 0.75 | (0.52–1.08) | 0.123 |
| Siglec-9 | 1.14 | (0.87–1.49) | 0.353 |
| SLAMF1 | 1.08 | (0.85–1.37) | 0.510 |
| SOX2 | 0.98 | (0.92–1.05) | 0.559 |

| | | | |
|---|---|---|---|
| Thyroglobulin | 1.14 | (0.99–1.32) | 0.074 |
| TLR4 | 1.12 | (0.99–1.27) | 0.063 |
| TPST-2 | 1.03 | (0.97–1.11) | 0.334 |
| VEGF-A | 1.16 | (0.94–1.42) | 0.157 |
| VEGF-D | 1.06 | (0.82–1.37) | 0.645 |

Eight proteins whose concentration were below LOD were excluded.

Abbreviations: DFS: disease-free survival; ShhN: Sonic Hedgehog N-Terminal. LOD: the lower limit of detection.

**Table S7. Univariate analysis of the 42 differently expressed proteins associated with overall survival.**

| | OS | | |
|---|---|---|---|
| | HR | 95% CI | *P* |
| Annexin A5 | 1.19 | (1.05–1.36) | 0.006 |
| BAFF-R | 1.11 | (0.90–1.35) | 0.328 |
| BID | 1.00 | (0.94–1.07) | 0.901 |
| CA 15-3 | 0.94 | (0.83–1.07) | 0.336 |
| Cadherin-4 | 1.06 | (0.86–1.32) | 0.584 |
| Calsyntenin-1 | 1.02 | (0.83–1.24) | 0.866 |
| CD155 | 0.97 | (0.87–1.08) | 0.580 |
| SLAMF5 | 1.31 | (1.06–1.63) | 0.014 |
| GST-2 | 1.06 | (0.97–1.15) | 0.233 |
| C-IAP2 | 1.01 | (0.86–1.18) | 0.890 |
| CK-18 | 1.05 | (0.98–1.13) | 0.153 |
| Delta1 | 1.05 | (0.88–1.24) | 0.599 |
| ESM-1 | 1.46 | (1.09–1.95) | 0.011 |
| EphA2 | 1.16 | (0.90–1.48) | 0.246 |
| ErbB4 | 1.07 | (0.86–1.33) | 0.531 |
| Ficolin-1 | 1.21 | (0.99–1.49) | 0.068 |
| FLT-3 | 1.00 | (0.87–1.15) | 0.999 |
| GH receptor | 1.12 | (0.88–1.42) | 0.376 |
| CXCL1 | 1.03 | (0.96–1.09) | 0.409 |
| IL-1F10 | 1.00 | (0.81–1.24) | 0.979 |
| IL-27RA | 1.27 | (0.92–1.76) | 0.149 |
| INSR | 1.19 | (1.02–1.39) | 0.023 |
| PSIP1 | 1.04 | (0.93–1.16) | 0.490 |
| MCP-2 | 1.37 | (1.00–1.86) | 0.048 |
| MMP-8 | 1.56 | (1.09–2.22) | 0.014 |
| Neurturin | 1.46 | (1.01–2.13) | 0.047 |
| NPDC-1 | 1.47 | (1.03–2.11) | 0.034 |
| PDGF-C | 1.12 | (0.96–1.32) | 0.153 |
| DLK-1 | 1.18 | (0.92–1.51) | 0.195 |
| Renin | 0.67 | (0.50–0.90) | 0.008 |
| Semaphorin-4C | 1.01 | (0.94–1.08) | 0.821 |
| Serpin A5 | 2.53 | (1.45–4.39) | 0.001 |
| Serpin F1 | 1.26 | (0.90–1.75) | 0.176 |
| ShhN | 0.61 | (0.39–0.94) | 0.027 |
| Siglec-9 | 1.17 | (0.83–1.66) | 0.374 |
| SLAMF1 | 0.97 | (0.72–1.31) | 0.863 |
| SOX2 | 0.98 | (0.90–1.06) | 0.603 |

| | | | |
|---|---|---|---|
| Thyroglobulin | 1.16 | (0.96–1.40) | 0.134 |
| TLR4 | 1.08 | (0.93–1.25) | 0.315 |
| TPST-2 | 1.02 | (0.94–1.11) | 0.584 |
| VEGF-A | 1.13 | (0.88–1.46) | 0.327 |
| VEGF-D | 1.10 | (0.81–1.50) | 0.548 |

Eight proteins whose concentrations were below LOD were excluded.

Abbreviations: OS: overall survival; ShhN: Sonic Hedgehog N-Terminal. LOD: the lower limit of detection.

**Table S8. The concentration of the 5 proteins of PSDM signature in high and low metastatic risk group stratified by the PSDM risk score.**

| | High-risk group (n = 81) | Low-risk group (n = 145) | |
|---|---|---|---|
| | Concentration (Mean ± SD) (log2 pg/ml) | Concentration (Mean ± SD) (log2 pg/ml) | ***P*** |
| SLAMF5 | 13.46 ± 1.05 | 12.30 ± 0.93 | <0.001 |
| ESM-1 | 8.81 ± 0.90 | 8.30 ± 0.62 | <0.001 |
| MMP-8 | 6.96 ± 0.55 | 5.62 ± 0.84 | <0.001 |
| INSR | 12.72 ± 2.12 | 10.90 ± 2.59 | <0.001 |
| Serpin A5 | 14.14 ± 0.53 | 13.61 ± 0.20 | <0.001 |

**Table S9. Five-year distant metastasis-free survival, disease-free survival and overall survival estimates for different groups.**

| 5-year survival | All patients (n = 226) | | Low EBV DNA load subgroup (n = 92) | | High EBV DNA load subgroup (n = 134) | |
|---|---|---|---|---|---|---|
| | Low risk (n = 145) | High risk (n = 81) | Low risk (n = 64) | High risk (n = 28) | Low risk (n = 81) | High risk (n = 53) |
| **DMFS (%)** | 94.4 | 64.2 | 95.1 | 85.7 | 93.8 | 52.8 |
| **(95% CI)** | (90.6–98.2) | (54.5–75.5) | (89.8–100) | (73.7–99.7) | (88.6–99.2) | (40.9–68.1) |
| **DFS (%)** | 81.9 | 58.0 | 82.5 | 78.6 | 81.3 | 47.1 |
| **(95% CI)** | (75.8–88.4) | (48.2–69.8) | (73.7–92.5) | (64.8–95.3) | (73.2–90.3) | (35.4–62.7) |
| **OS (%)** | 90.2 | 74.1 | 92.1 | 85.7 | 88.8 | 67.9 |
| **(95% CI)** | (85.5–95.2) | (65.1–84.2) | (85.6–99.0) | (73.7–99.7) | (82.1–96.0) | (56.5–81.7) |

Abbreviations: DMFS: distant metastasis-free survival; OS: overall survival; DFS: disease-free survival; CI: confidence interval; EBV DNA: Epstein-Barr virus DNA.

**Table S10. The number of events for different groups.**

| Number of events | All patients (n = 226) | | Low EBV DNA load subgroup (n = 92) | | High EBV DNA load subgroup (n = 134) | |
|---|---|---|---|---|---|---|
| | Low risk (n = 145) | High risk (n = 81) | Low risk (n = 64) | High risk (n = 28) | Low risk (n = 81) | High risk (n = 53) |
| Distant metastasis | 11 | 30 | 4 | 4 | 7 | 26 |
| Disease progression | 29 | 36 | 12 | 6 | 17 | 30 |
| Death | 17 | 25 | 7 | 4 | 10 | 21 |

Abbreviations: EBV DNA: Epstein-Barr virus DNA.

**Table S11. Clinicopathological characteristics of the 84 patients selected by propensity score matching in TPF IC benefit subgroup analysis.**

| | CCRT plus TPF-IC N(%) | CCRT alone N(%) | *P* |
|---|---|---|---|
| **Total population** | 42 | 42 | |
| **Age** | | | 0.99 |
| < 45 years | 23 | 24 | |
| ≥ 45 years | 19 | 18 | |
| **Gender** | | | 0.35 |
| Male | 38 | 34 | |
| Female | 4 | 8 | |
| **T Stage** | | | 0.40 |
| T1–2 | 10 | 6 | |
| T3–4 | 32 | 36 | |
| **N Stage** | | | 0.99 |
| N0–1 | 16 | 16 | |
| N2–3 | 26 | 26 | |
| **EBV DNA load (copies/mL)** | | | 0.50 |
| < 2000 | 19 | 15 | |
| ≥ 2000 | 23 | 27 | |
| **Radiotherapy** | | | 0.99 |
| 3D-CRT or IMRT | 42 | 42 | |

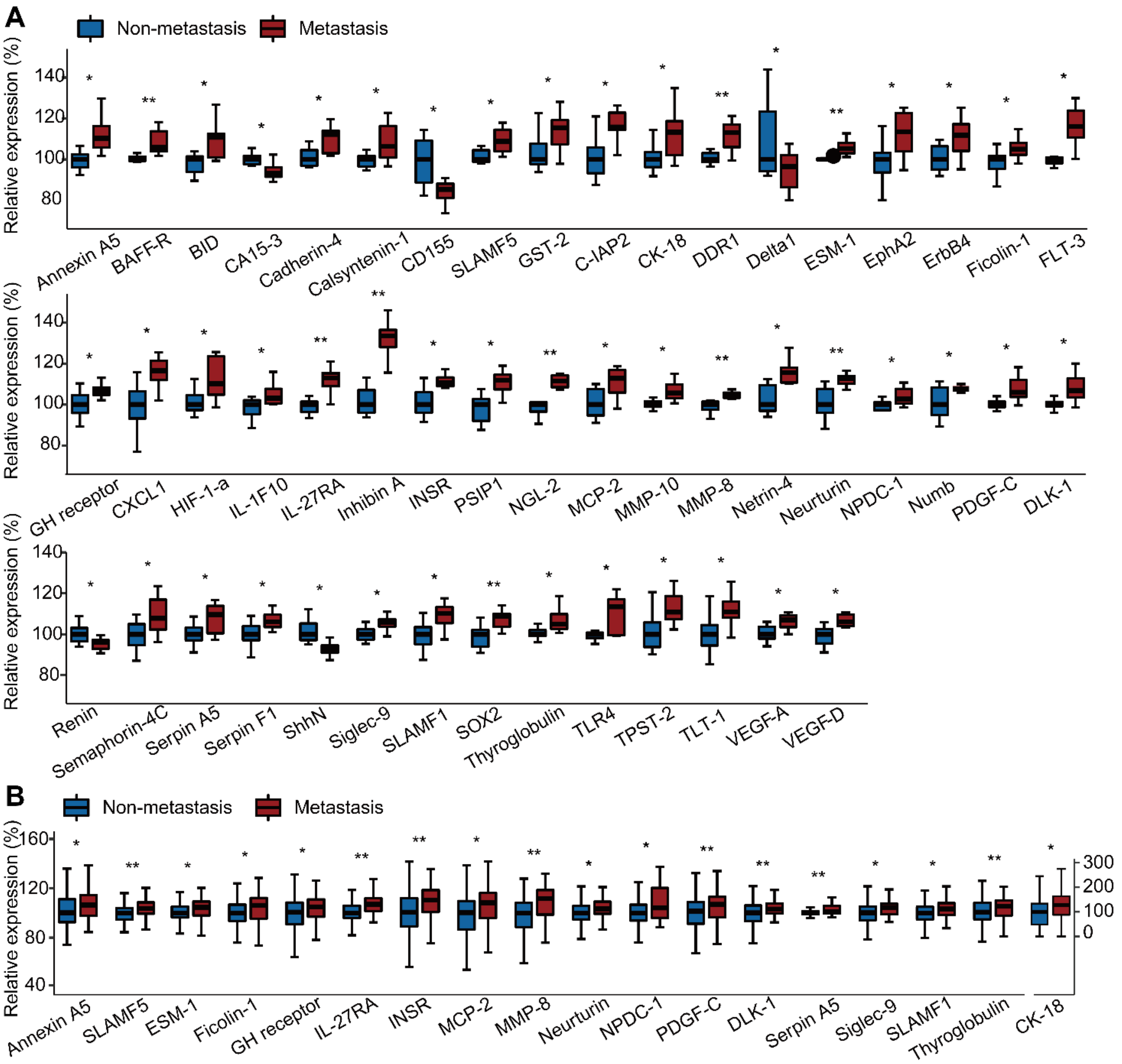


**Figure S1. Expression of plasma proteins related to metastasis in the high-throughput and low-throughput arrays.**

(A) The 50 differentially expressed proteins discovered using the high-throughput antibody arrays. Student's t-test, * $P < 0.05$, ** $P < 0.01$ (B) The 18 proteins that differentially expressed between metastatic and non-metastatic NPC patients using the low-throughput customized quantitative antibody arrays. Student's t-test, * $P < 0.05$, ** $P < 0.01$. The expression values were log2-transformed.

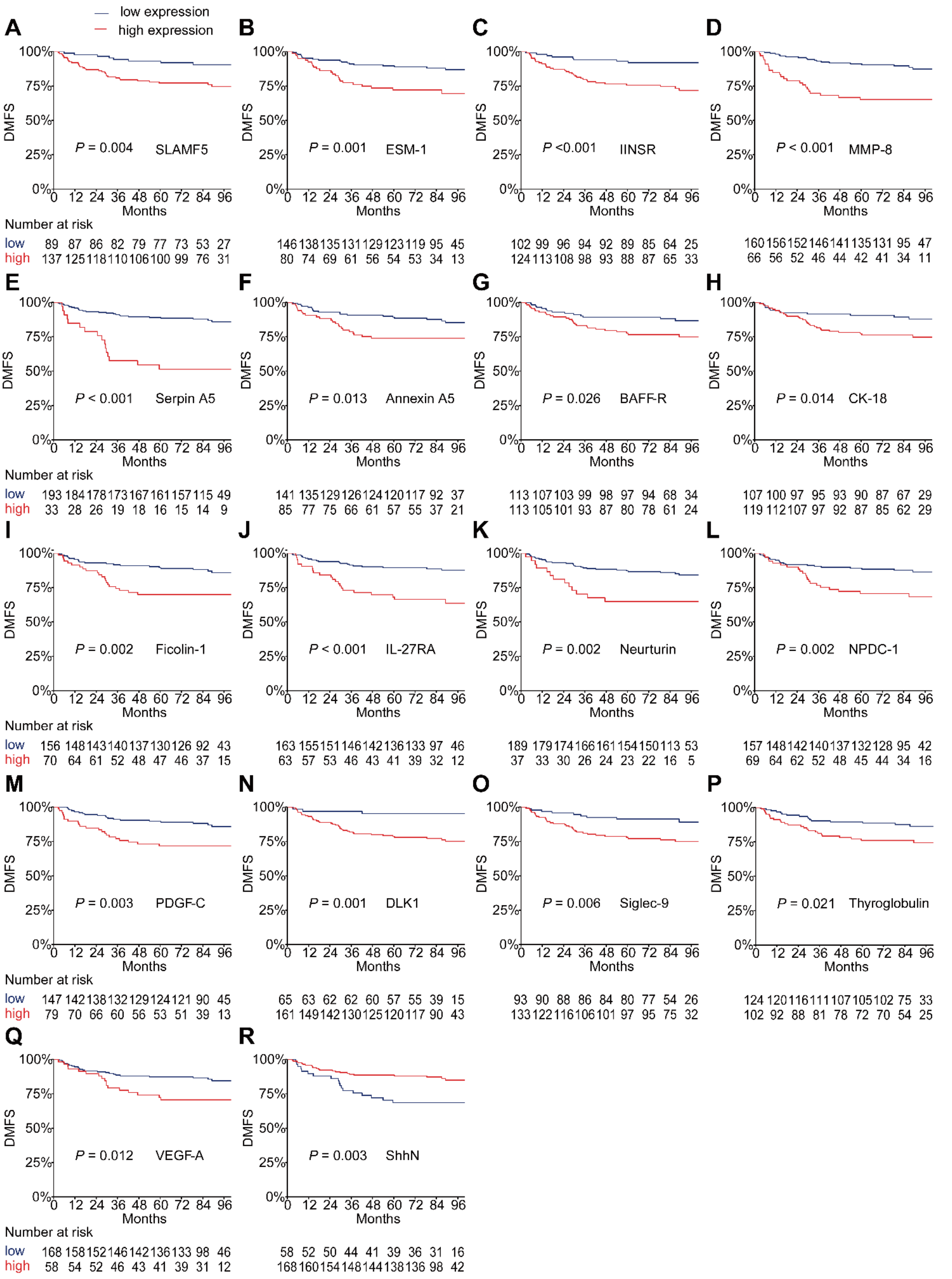


**Figure S2. Kaplan–Meier curves of distant metastasis-free survival (DMFS) according to the 18 proteins associated with DMFS.**

Plots show (A) SLAMF5; (B) ESM-1; (C) INSR; (D) MMP-8; (E) Serpin A5; (F) Annexin A5; (G) BAFF-R; (H) CK-18; (I) Ficolin-1; (J) IL-27RA; (K) Neurturin; (L) NPDC-1; (M) PDGF-C; (N)DLK1; (O) Siglec-9; (P) Thyroglobulin; (Q) VEGF-A; (R) ShhN.

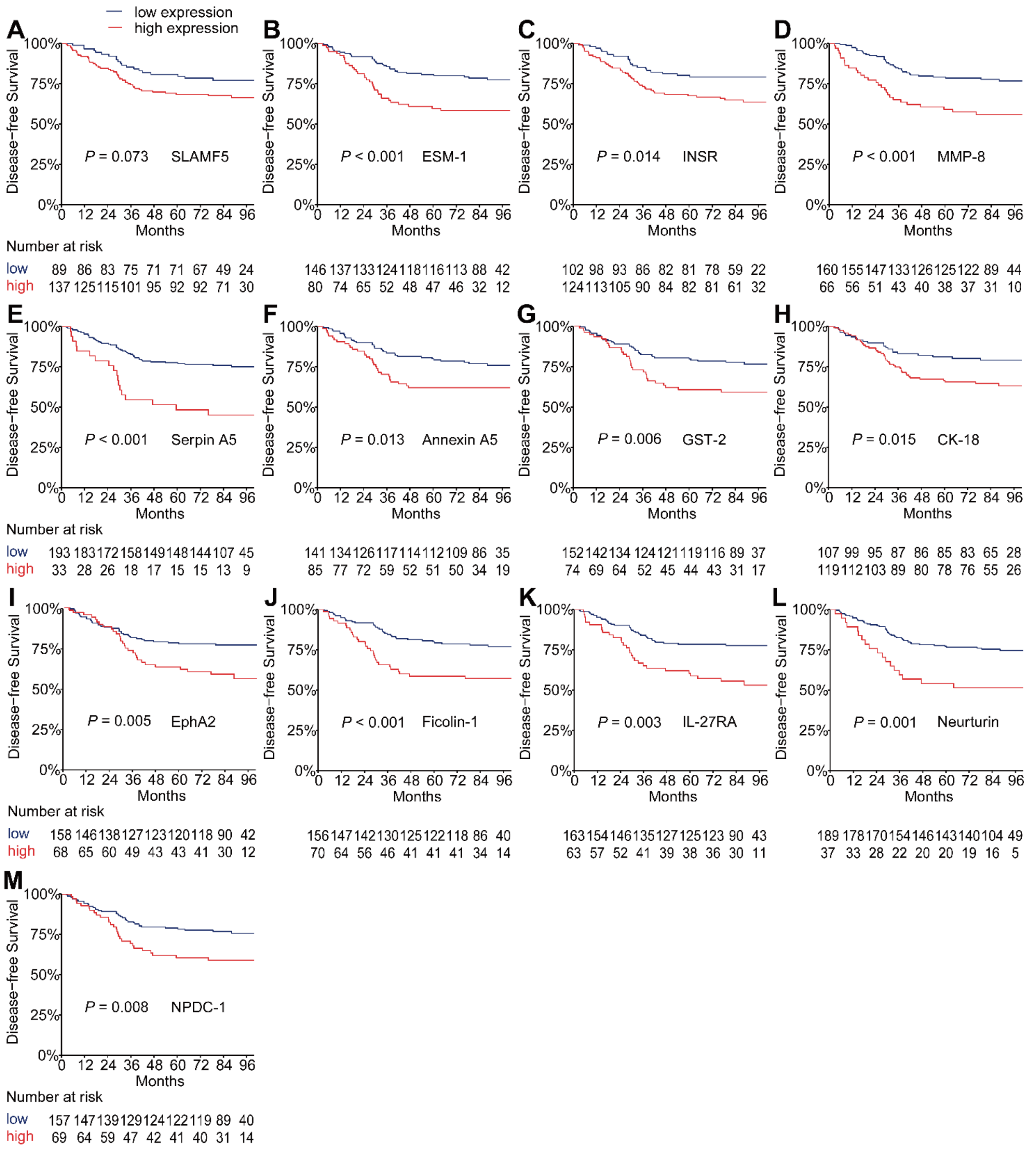


**Figure S3. Kaplan–Meier curves of disease-free survival (DFS) according to the 13 proteins associated with DFS.**

Plots show (A) SLAMF5; (B) ESM-1; (C) INSR; (D) MMP-8; (E) Serpin A5; (F) Annexin A5; (G) GST-2; (H) CK-18; (I) EphA2; (J) Ficolin-1; (K) IL-27RA; (L) Neurturin; (M) NPDC-1.

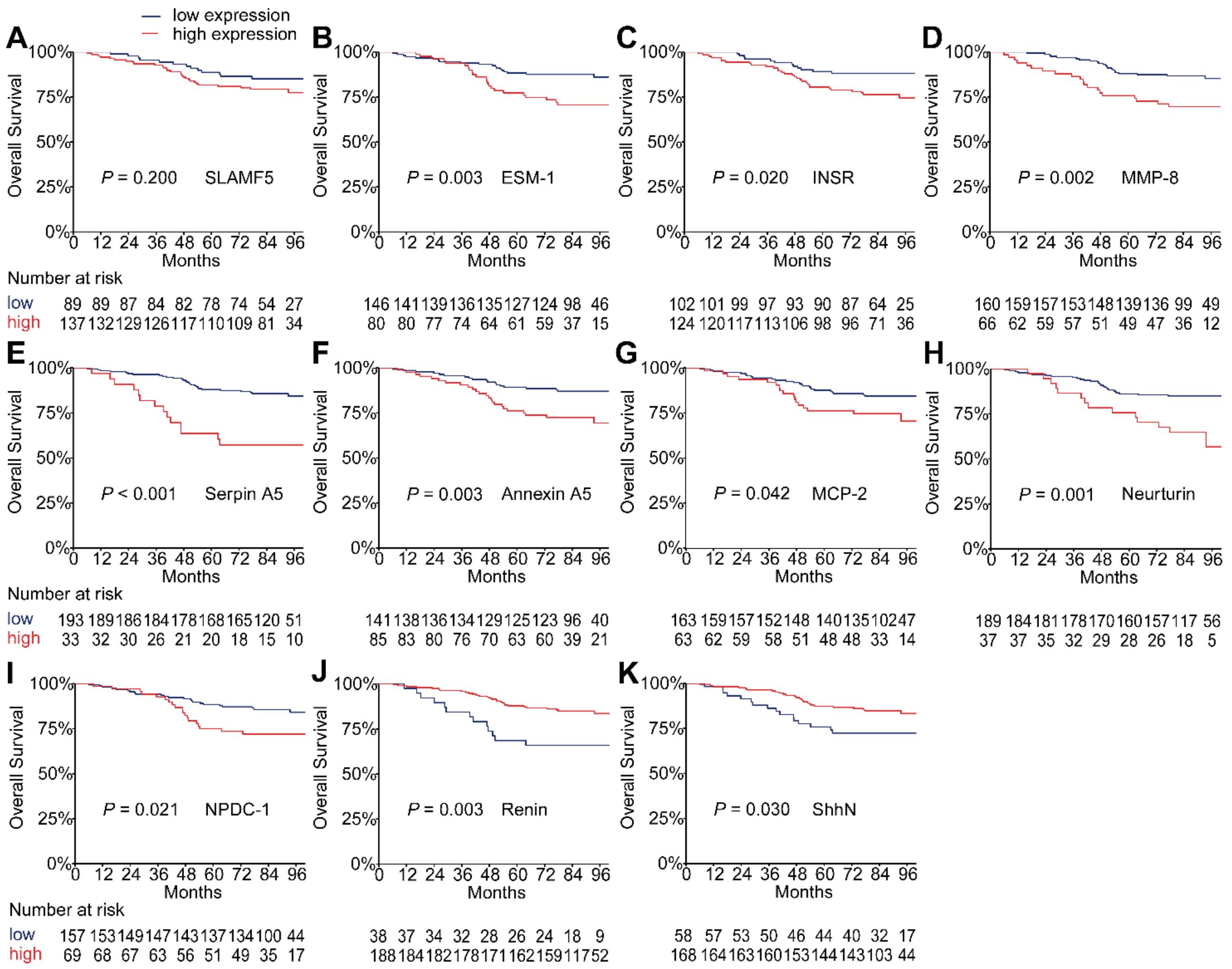


**Figure S4. Kaplan–Meier curves of overall survival (OS) according to the 11 proteins associated with OS.**

Plots show (A) SLAMF5; (B) ESM-1; (C) INSR; (D) MMP-8; (E) Serpin A5; (F) Annexin A5; (G) MCP-2; (H) Neurturin; (I) NPDC-1; (J) Renin; (K) ShhN.

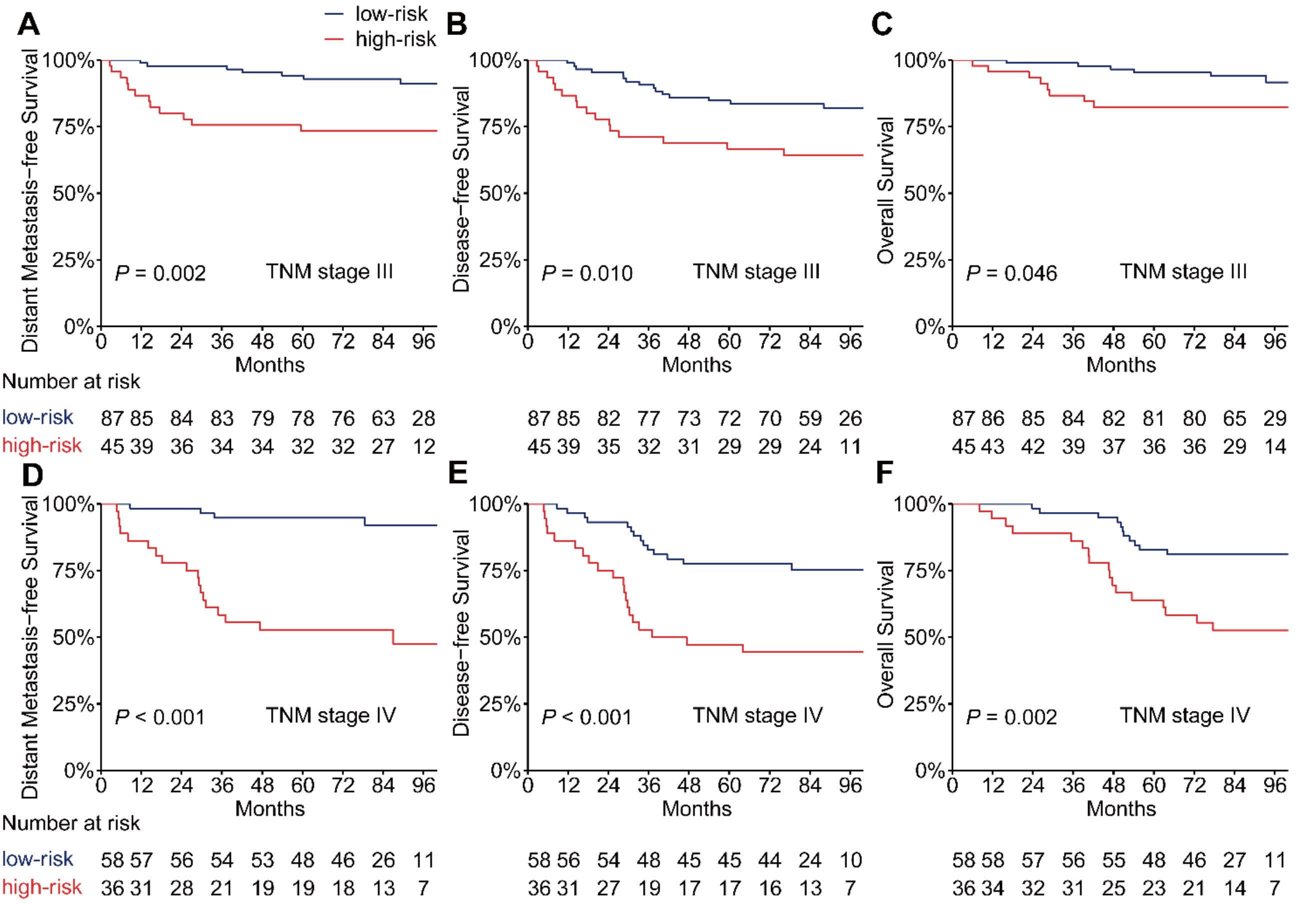


**Figure S5. Kaplan–Meier survival curves for patients with low risk and high risk according to the PSDM in stage III or stage IV subgroup.**

Plots show (A) distant metastasis-free survival, (B) disease-free survival and (C) overall survival for NPC patients in the stage III subgroup. (D) distant metastasis-free survival, (E) disease-free survival and (F) overall survival for NPC patients in the stage IV subgroup. Abbreviations: PSDM: protein-based signature for distant metastasis. HR: hazard ratio; and CI: confidence interval.

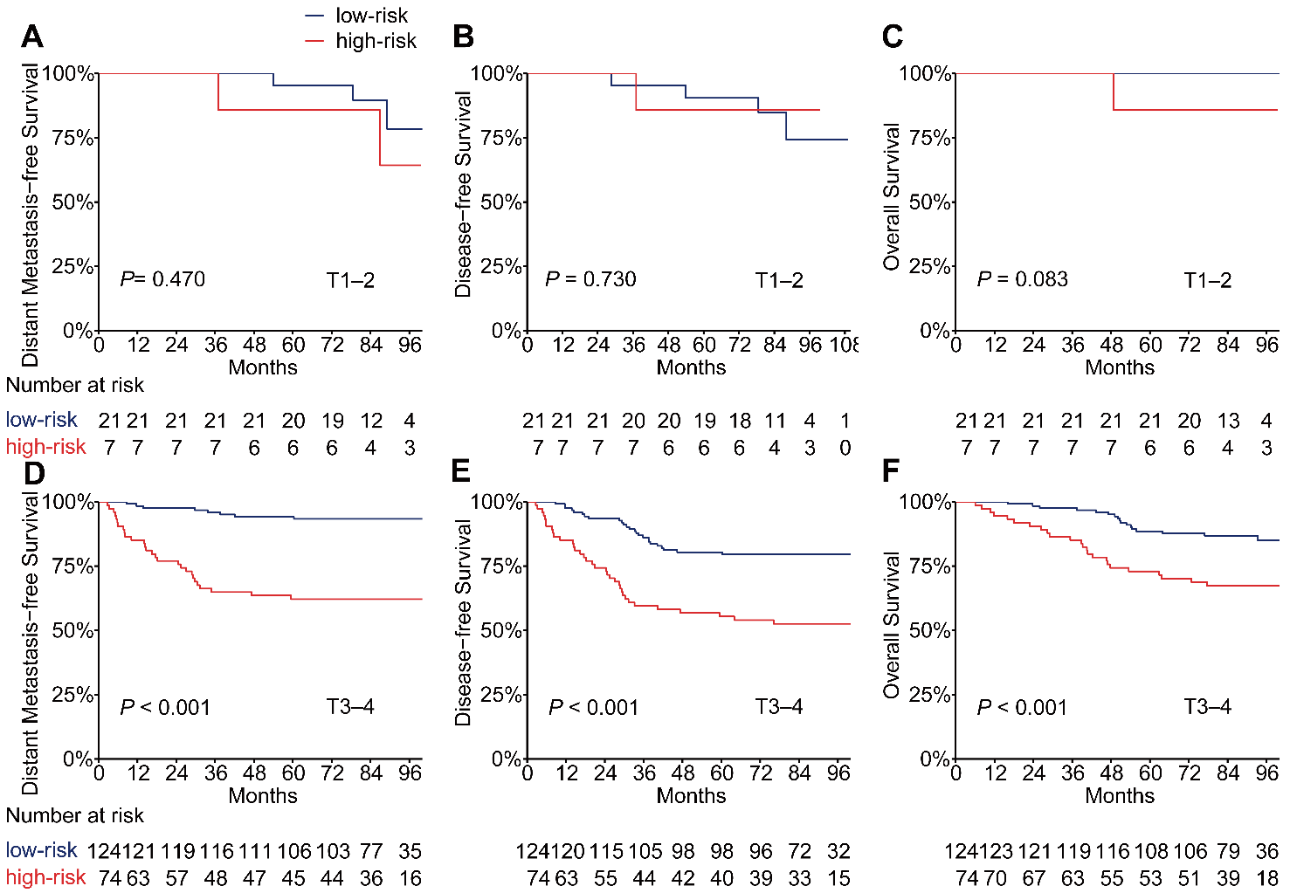


**Figure S6. Kaplan–Meier survival curves for patients with low risk and high risk according to the PSDM in T1–2 or T3–4 subgroup.**

Plots show (A) distant metastasis-free survival, (B) disease-free survival and (C) overall survival for NPC patients in the T1–2 subgroup. (D) distant metastasis-free survival, (E) disease-free survival and (F) overall survival for NPC patients in the T3–4 subgroup. Abbreviations: PSDM: protein-based signature for distant metastasis. HR: hazard ratio; and CI: confidence interval.

(There were only 28 patients in the T1–2 subgroup and no patient died of NPC in the low-risk group, which may lead to bias.)

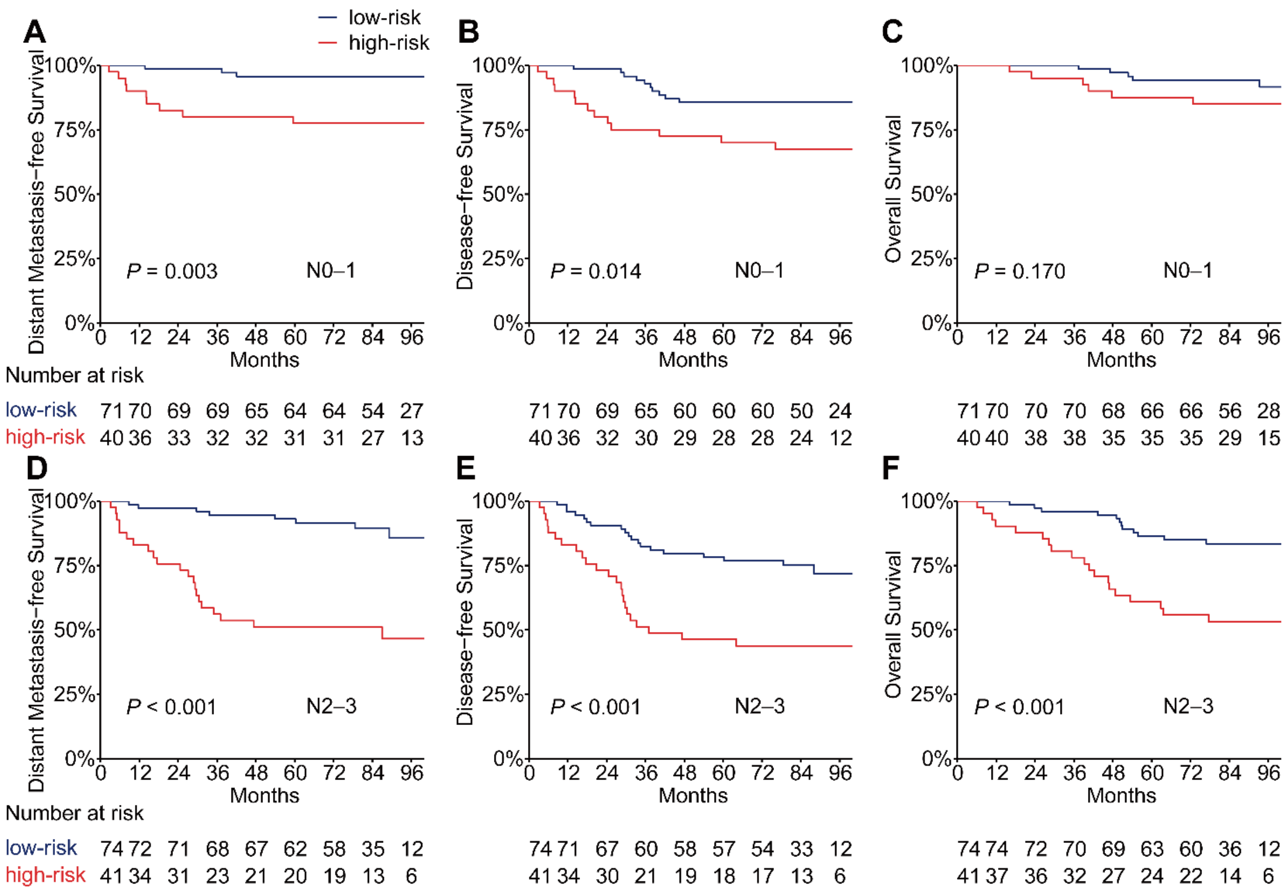


**Figure S7. Kaplan–Meier survival curves for patients with low risk and high risk according to the PSDM in N0–1 or N2–3 subgroup.**

Plots show (A) distant metastasis-free survival, (B) disease-free survival and (C) overall survival for NPC patients in the N0–1 subgroup. (D) distant metastasis-free survival, (E) disease-free survival and (F) overall survival for NPC patients in the N2–3 subgroup. Abbreviations: PSDM: protein-based signature for distant metastasis. HR: hazard ratio; and CI: confidence interval.

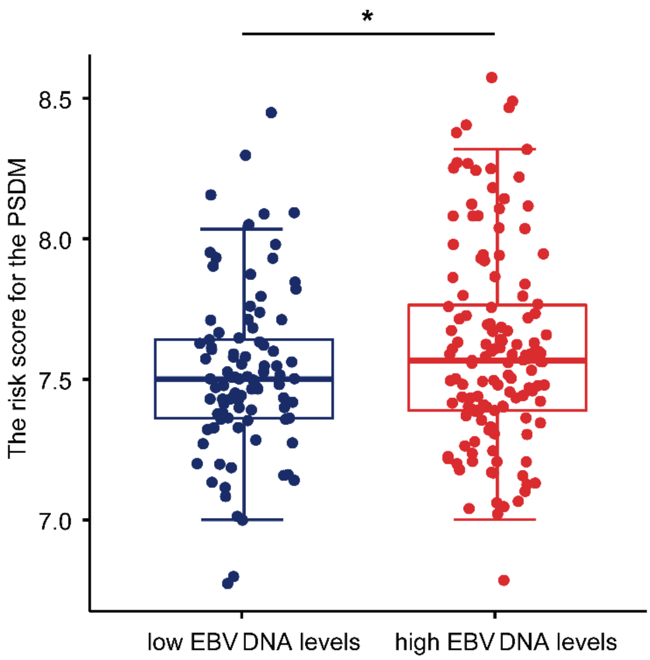


**Figure S8. The risk scores for PSDM in the low and high EBV DNA subgroups.**
